# Two factor-based reprogramming of rodent and human fibroblasts into Schwann cells

**DOI:** 10.1038/ncomms14088

**Published:** 2017-02-07

**Authors:** Pietro Giuseppe Mazzara, Luca Massimino, Marta Pellegatta, Giulia Ronchi, Alessandra Ricca, Angelo Iannielli, Serena Gea Giannelli, Marco Cursi, Cinzia Cancellieri, Alessandro Sessa, Ubaldo Del Carro, Angelo Quattrini, Stefano Geuna, Angela Gritti, Carla Taveggia, Vania Broccoli

**Affiliations:** 1 Division of Neuroscience, San Raffaele Scientific Institute, 20132 Milan, Italy; 2 University of Milano-Bicocca, 20126 Milan, Italy; 3 Division of Neuroscience, Axo-glia Interactions Unit-INSPE, San Raffaele Institute, 20132 Milan, Italy; 4 Department of Clinical and Biological Sciences, and Cavalieri Ottolenghi Neuroscience Institute, University of Turin, Ospedale San Luigi, 10043 Orbassano, Turin, Italy; 5 SR-Tiget, Division of Regenerative Medicine, Stem Cells and Gene Therapy, San Raffaele Scientific Institute, 20132 Milan, Italy; 6 Department of Neurology, Neurophysiology and Neurorehabilitation Unit-INSPE, San Raffaele Scientific Institute, 20132 Milan, Italy; 7 Department of Neurology, Neuropathology Unit-INSPE, San Raffaele Scientific Institute, Via Olgettina 48, 20132 Milan, Italy; 8 National Research Council (CNR), Institute of Neuroscience, 20129 Milan, Italy

## Abstract

Schwann cells (SCs) generate the myelin wrapping of peripheral nerve axons and are promising candidates for cell therapy. However, to date a renewable source of SCs is lacking. In this study, we show the conversion of skin fibroblasts into induced Schwann cells (iSCs) by driving the expression of two transcription factors, Sox10 and Egr2. iSCs resembled primary SCs in global gene expression profiling and PNS identity. *In vitro*, iSCs wrapped axons generating compact myelin sheaths with regular nodal structures. Conversely, iSCs from Twitcher mice showed a severe loss in their myelinogenic potential, demonstrating that iSCs can be an attractive system for *in vitro* modelling of PNS diseases. The same two factors were sufficient to convert human fibroblasts into iSCs as defined by distinctive molecular and functional traits. Generating iSCs through direct conversion of somatic cells offers opportunities for *in vitro* disease modelling and regenerative therapies.

## Abstract (web summary)

Schwann cells (SCs) myelinate peripheral nerve axons and offer opportunities for the treatment of injuries and demyelinating diseases but reliable and renewable sources of these cells are hard to come by. Here the authors reprogram rat, mouse and human fibroblasts into Schwann cells using two transcription factors.

Schwann cells (SCs) are neural crest-derived cells able to produce the myelin sheaths, wrapping neuronal axons in the peripheral nervous system (PNS). When peripheral nerves are injured, SCs adaptively respond by supporting and stimulating tissue regeneration^1^. Nevertheless, after severe nerve injuries or in genetic and metabolic myelin disorders, the loss of myelin ensheathing axons cannot be replaced, leading to disabling sensory defects and motor dysfunctions^2^,^3^. A valuable therapeutic option for the treatment of peripheral nerve insults is represented by the transplantation of SCs, alone or in combination with the nerve guide^4^,^5^. However, this therapeutic approach is strongly limited by the current lack of a renewable source of SCs in humans.

Isolation of primary cultures of myelin-competent SCs works very poorly in mice and humans^6^ and methods currently available for differentiating SCs from pluripotent stem cells are time-consuming, technically complex and generate SC precursors with unproven myelination potential^7^. Generation of SCs has been recently obtained through differentiation of somatic progenitor cells^8^,^9^. Nonetheless, these approaches are limited by the need of isolating rare progenitor cells in tissues. Moreover, most of these methods are laborious and generate SCs with low myelination efficiency that strongly limits the development of cell-based therapies and *in vitro* disease-modelling studies. To overcome these limitations, we speculated that a direct cell conversion approach to convert skin fibroblasts into SCs would offer a more straightforward and convenient procedure. Supra-physiological expression of defined sets of developmental neural transcription factors (TFs) is sufficient to impose a neural identity to somatic cells in a rapid and single-step procedure, generating induced neurons and glial cells with mature morphological and functional properties^10^,^11^,^12^,^13^,^14^. In particular, TF-mediated reprogramming can be applied to generate induced oligodendrocyte precursor cells that express appropriate OPC markers, produce myelin sheaths *in vitro* and sustain myelin regeneration in mouse brains with genetic dysmyelination^15^,^16^. Importantly, induced oligodendrocyte precursor cells were shown to lack Myelin protein zero (MPZ) protein, a specific SC marker, and myelinated multiple axons confirming their central, and not peripheral, glial cell identity^15^,^16^. We, therefore, sought to determine whether SCs could be generated by direct lineage conversion from readily available somatic lineages such as fibroblasts. We identified two factors sufficient to convert rodent fibroblasts into SCs with molecular PNS identity and competent to generate compact and functional myelin sheets. The same factor combination could be used to promote conversion of human post-natal fibroblasts into SCs with comparable properties and functions.

## Results

### Two TF-based reprogramming of fibroblasts into SCs

**Figure 1:**
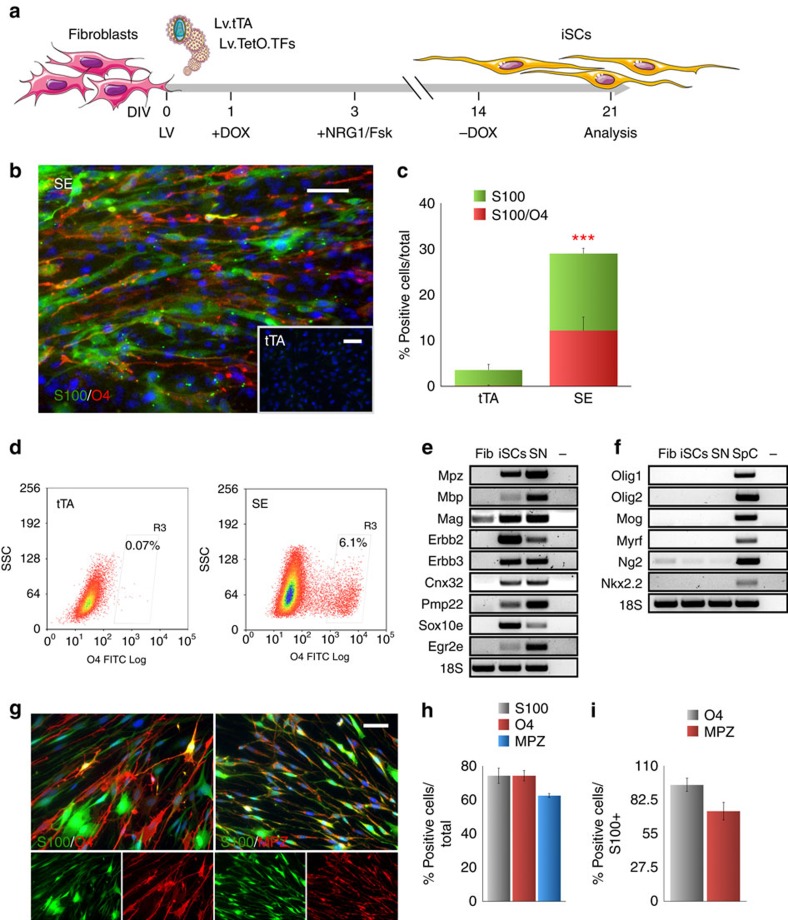
Direct conversion of mouse skin fibroblasts into iSCs. (**a**) Schematic representation of the reprogramming strategy for generating iSCs using the dox-inducible lentiviral transgenes expressing tTA, combinations of transcription factors (TFs) together with the small molecules NRG1 and Fsk. (**b**) S100 (green) and O4 (red) immunofluorescence staining of Sox10/Egr2 (SE) or tTA-infected (inset) MEFs. (**c**) Quantification of S100^+^ and S100^+^/O4^+^ cells expressed as percentage over the total number of cells in experimental (SE) and control (tTA) conditions at 14 DIV (Mean±s.d., *n*=8 independent experiments, 10 randomly selected 20 × fields per sample were examined). (**d**) Fluorescence-activated cell sorting-based purification of the O4^+^ iSC population. (**e**) Expression of SC cardinal markers by RT–PCR in fibroblasts (Fib), induced SCs and adult sciatic nerve (SN, positive control). Sox10e, endogenous Sox10, Egr2e, endogenous Egr2. (**f**) Expression of Oligodendrocyte-specific markers by RT–PCR in fibroblasts (Fib), induced SCs, sciatic nerve (SN) and P20 spinal cord (SpC, positive control). (**g**) S100/O4 and S100/MPZ immunofluorescence staining in iSCs. (**h**,**i**) Quantification of 14 DIV iSCs expressing S100, O4 and MPZ alone or in combination (Mean±s.d., *n*=6 independent experiments, three coverslips/experiment/antigen, 10 randomly selected 20 × fields per sample were examined). ***P<0,001; Student's *t*-test. Scale bars, 50 μm.

Over the last decade, an intertwined regulatory network has been shown to have a critical role in promoting PNS myelination and its maintenance^17^,^18^. We selected Sox10, Pou3f1 (known also as Oct6), Egr2 (known also as Krox20) and Brn2 for their cardinal role during SC myelination as good candidates for cell lineage reprogramming^19^. To this end, the factors were individually cloned in doxycycline (dox)-inducible lentiviral vectors and E15.5 mouse embryonic fibroblasts were infected with one or more lentiviruses and cultured in a SC culture medium supplemented with Neuregulin-1 (NRG1) and forskolin (Fsk) (Fig. 1a)^20^. At first, we realized that primary cultures of embryonic and adult skin fibroblasts often contain a fraction of CD271^+^ cells with neural crest stem cell features and can give rise to SC precursors (Supplementary Fig. 1a,b)^21^. Thus, before each reprogramming experiment, primary fibroblast cultures were selected against CD271^+^ cells by flow-cytometry with a stringent gating selection (Supplementary Fig. 1c). To evaluate the SC lineage conversion, we monitored for the concomitant activation of S100 and O4, both highly expressed in SCs, likely in an immature state, while undetectable in fibroblasts. Fourteen days *in vitro* (DIV) after viral-mediated gene transfer the amount of double S100/O4 positive cells was scored with the different TF combinations (Supplementary Fig. 1d,e). Interestingly, we found that Sox10 combined with Egr2 expression produced the highest amount of S100^+^/O4^+^ cells (12.3±3.1% of total population, *n*=8 independent experiments. Values are means±s.d.) (Fig. 1b,c and Supplementary Fig. 1d,e). The eventual addition of either Pou3f1 or Brn2 as a third factor did not further increase the yield of S100/O4 expressing cells (Supplementary Fig. 1e). Conversely, tTA infected or not infected (control) fibroblasts did not show detectable expression of either SC marker (Fig. 1b, inset). To better examine the reprogrammed cell fraction, we isolated the O4^+^ cell population by flow-cytometry (Fig. 1d) and expanded the culture for the following analysis. Candidate gene expression analysis of the Sox10/Egr2 reprogrammed cell progeny revealed a strong activation of several SC markers, such as Erbb2, Erbb3, Cnx32, Pmp22 and Mpz (also known as P0) (Fig. 1e)^22^. Likewise, Mpz protein was readily detectable by immunostaining in the great majority of the S100^+^/O4^+^ cellular fraction as it is normally found in somatic peripheral SCs, but not in CNS oligodendrocytes (Fig. 1g–i)^22^. Conversely, sorted cells did not express oligodendrocytes-specific transcription factors and markers such as Olig1 and Olig2, Mog, Myrf (Mrf), Ng2 and Nkx2.2 (Fig. 1f) confirming their PNS regional identity. In addition, endogenous Sox10 and Egr2 were expressed in transduced fibroblasts indicating that crucial components of the SC gene regulatory network were reactivated in the Sox10/Egr2, but not tTA, infected cells. (Fig. 1e). In accordance, known direct target genes of Sox10 and Egr2 during SC development and myelination such as Prx, Erbb3 and Mpz, were strongly activated as confirmed by quantitative reverse transcription PCR (qRT–PCRs) (Supplementary Fig. 1f)^23^. Importantly, silencing of the two lentiviral transgenes by dox withdrawal for 2 weeks did not alter the expression of S100 and O4, indicating a stable expression of these SC markers independently by the reprogramming process (Supplementary Fig. 1g). Thus, given the strong activation of the crucial factors distinctive for the SC lineage and their subsequent maintenance even after transgene expression silencing, we named the Sox10/Egr2 reprogrammed fibroblasts as induced Schwann cells (iSCs). The two factors were also effective in reprogramming neonatal and adult murine skin fibroblasts generating S100^+^/O4^+^ iSCs with an efficiency of 8.5±3.1% and 18.9±5.3% of total population, respectively (*n*=4) (Supplementary Fig. 2). Despite the driving force of the reprogramming factors, the culture conditions proved to be critical for the subsequent survival of the reprogrammed cells. In particular the depletion of both NRG1 and Fsk, but not Fsk alone, caused a strong reduction in the number of S100/O4/Mpz^+^ iSCs due to a significant increase in cell death (Supplementary Fig. 3).

### Transcriptional profiling of iSCs

Given that the establishment of pure and highly proliferating SC cultures is often obtained starting from neonatal rats, we wondered whether our method could be applied in other species rather than the mouse. Rat neonatal fibroblasts were converted into iSCs by the two-factor expression with similar efficiency and dynamics compared to mouse cells (Supplementary Fig. 4a,b). Fluorescence-activated cell sorting-isolated rat O4^+^ iSCs showed an elongated bipolar shape and resulted immunodecorated for S100 and Mpz in most cases (Supplementary Fig. 4c–e). Thus, *in vitro* cultures of purified rat O4^+^ iSCs, their respective fibroblasts of origin and somatic SCs were profiled for genome-wide expression by next-generation sequencing analysis of total RNA (RNA-seq).

**Figure 2:**
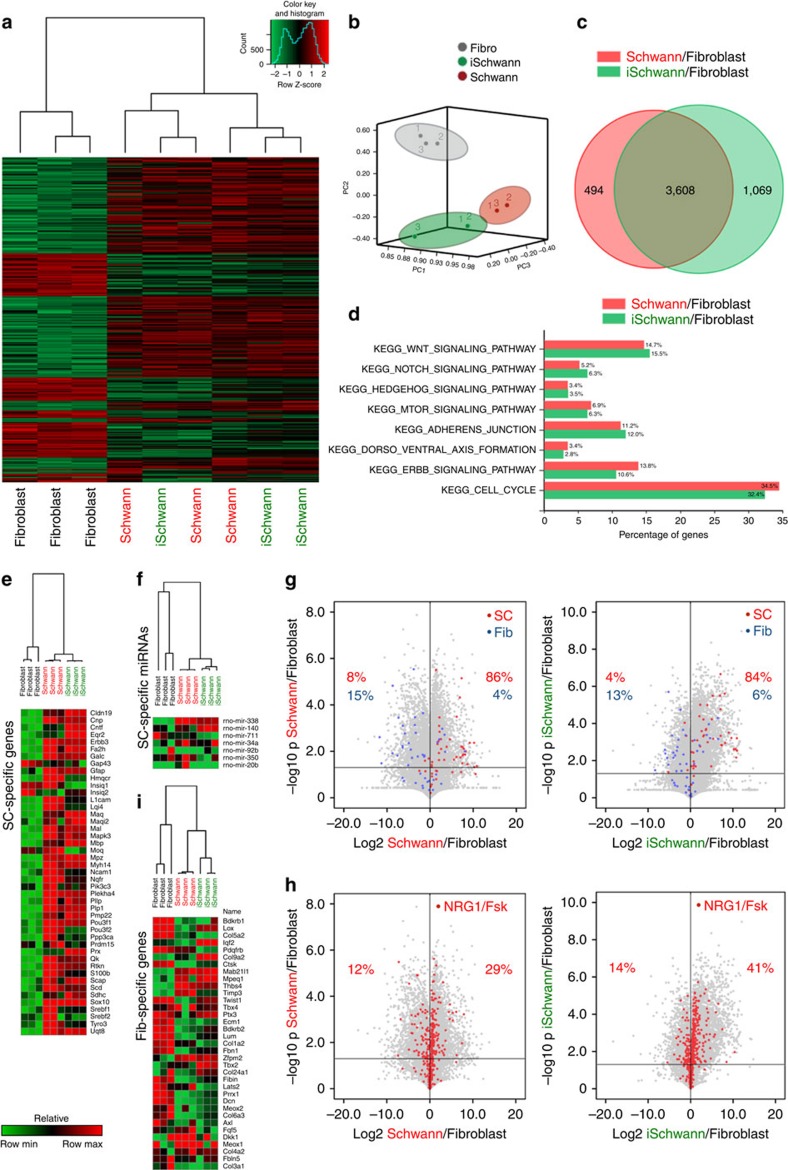
Comparative transcriptomic analysis of iSCs, primary SCs and fibroblasts of origin. (**a**) Heat-map of the unsupervised hierarchical clustering (UHC) of 2,500 random genes for fibroblasts and somatic and induced Schwann cells. The correlation between samples is shown as a dendrogram on top of the picture. (**b**) Principal component analysis showing the first three components, explaining about 75% of the total variance. (**c**) Venn diagram showing the overlap between the differentially expressed genes (DEGs) comparing Schwann and iSchwann cells versus fibroblasts. (**d**) Functional enrichment analysis of the DEGs. (**e**,**f**) UHC of somatic SC-specific genes/microRNAs in the three cell populations. (**g**) Volcano plots showing the distribution of SC- and fibroblast-specific genes in the two conditions. (Chi-square test SC-specific genes *P*=0.94; Chi-square test fibroblast-specific genes *P*=0.81) (**h**) Volcano plots showing the distribution of the genes perturbed upon exposure to NRG1/Fsk in the two comparisons (Chi-square test *P*=0.82). (**i**) UHC of fibroblast-specific genes in the three cell populations.

Remarkably reprogrammed and somatic SCs, but not fibroblasts, display strong correlation as assessed by unsupervised hierarchical clustering (UHC), principal component analysis and correlation of gene expression patterns (Fig. 2a,b and Supplementary Fig. 5a). Differential gene expression analysis showed about 4,000 transcripts differentially expressed between either iSCs or SCs versus fibroblasts. Of note, a large set of the differentially expressed genes (DEGs) was shared by the two SC populations (Fig. 2c). Moreover, gene ontology analysis revealed functional enrichment for multiple biological pathways invaluable for SC biology (Fig. 2d). Furthermore, by considering only those genes/microRNAs known to be pivotal for SC development, UHC clustered together iSCs and somatic SCs, thus confirming the similarities between the two cell types (Fig. 2e,f). Accordingly, qRT–PCR analysis confirmed that both SC derivatives showed comparable levels of many SC-enriched transcripts, including Cnp, Erbb3, GalC, Mal, Mapk3, Mbp, Mpz, Pmp22 and Pou3f1 (Supplementary Fig. 5b). To corroborate the similarities between the two SC types, we filtered for those genes physiologically relevant in SCs during development^24^ and subdivided SC- and fibroblast-specific DEGs according to fold change and statistical significance. Noteworthy, analysis of the DEG frequency distribution did not show any deviation of the observed frequencies (iSCs) from the expected frequencies (SCs) neither in SC- and fibroblast-specific DEGs, nor in SC developing genes (Fig. 2g and Supplementary Fig. 5d). Similarly, analysis of the DEG frequency distribution of those genes perturbed upon exposure to the differentiating factors NRG1 and Fsk did not show any deviation of the DEG frequencies (Fig. 2h)^25^. UHC filtering for genes belonging to biological pathways relevant for SC biology revealed an extensive clustering between iSCs and SCs (Supplementary Fig. 5e–j). Conversely, iSCs showed a consistent silencing of the genes specifically enriched in the fibroblasts. In fact, UHC analysis of a representative array of fibroblast-specific genes revealed that the majority of these were either strongly downregulated or even completely silenced in iSCs (Fig. 2i). These results were corroborated by qRT–PCR assays showing that somatic and induced SCs exhibited comparable levels of expression for randomly selected genes such as Col2a1, Col5a2, Ecm1 and Prrx1 (Supplementary Fig. 5c). Of note, only a minimal fraction of this class of genes (<5%), normally very poorly detectable in somatic SCs, were still expressed in 3-weeks reprogrammed iSCs although to a reduced extent compared with fibroblasts (Supplementary Fig. 5c). These results show that iSCs do not represent a hybrid state of SCs and the donor cell type, but instead exhibit a global transcriptional reconfiguration towards the reprogrammed cell type with minimal signs of memory at gene expression level of the cells of origin.

### bFGF is a potent mitogen for iSCs

**Figure 3:**
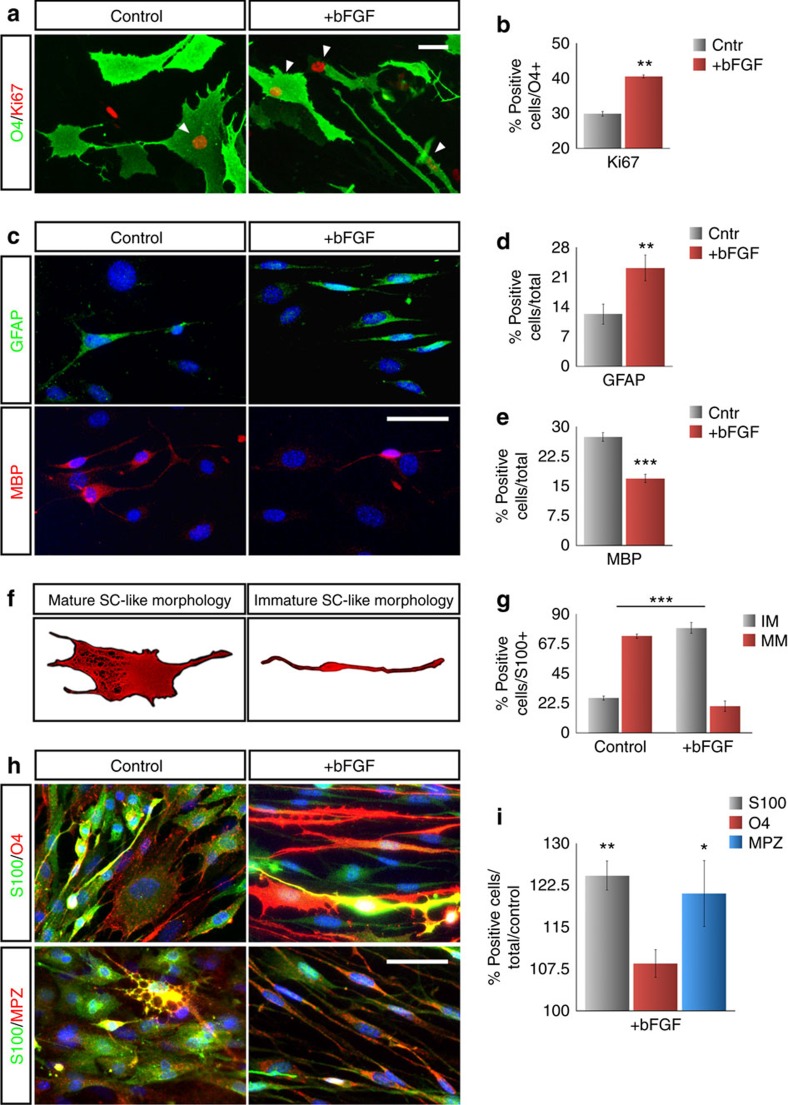
bFGF maintains iSCs in an immature and highly proliferative state. Immunofluorescence (**a**) and quantification (**b**) for Ki67 staining in O4^+^ iSCs with or without bFGF stimulation 1 week after dox withdrawal (Mean±s.d., *n*=5 independent experiments, three coverslips for each, 10 randomly selected 20 × fields per sample were examined). Immunofluorescence (**c**) and quantification (**d**,**e**) for GFAP and MBP staining in the presence or not of bFGF (Mean±s.d., *n*=3 independent experiments, two coverslips/experiment/antigen, 10 randomly selected 20 × fields per sample were examined). (**f**) Schematic view of a single SC with a mature (left) or immature (right) morphology. (**g**) Quantification of S100^+^ iSCs with mature (MM) and immature (IM) morphology in the two bFGF conditions (Mean±s.d., *n*=3 independent experiments, three coverslips/experiment/antigen, 10 randomly selected 20 × fields per sample were examined). Immunofluorescence (**h**) and quantification (**i**) for S100/O4 and S100/MPZ stainings in iSCs at week 1 after dox withdrawal in the absence (left) or presence (right) of bFGF (Mean±s.d., *n*=3 independent experiments, 10 randomly selected 20 × fields per sample were examined). *P<0,05; **P<0,01; ***P<0,001; Student's *t*-test (**b**,**d**,**e**); One-way ANOVA (**g**); Multiple *t*-test (**i**). Scale bars, 50 μm.

We, next, asked whether additional molecules added during reprogramming could further improve iSCs properties. We selected VEGF, basic fibroblast growth factor (bFGF) and TGFβ for their known role during Schwann cell commitment and maturation^26^. Whereas with VEGF and TGFβ we did not observe any evident effect, in the presence of bFGF, iSCs displayed a significant increase in cell proliferation as shown by an increased number of Ki67^+^ cells within the O4^+^ cell population (Fig. 3a,b). In addition, bFGF treatment promoted a sustained proliferation rate over time in iSC cultures (Supplementary Fig. 6). Importantly, bFGF-treated cells strongly upregulated GFAP, a molecular marker of immature SCs while significantly repressing the myelinating-associated protein Mbp (Fig. 3c–e). In accordance, bFGF sustained a global change in iSC morphology from mature (with extensive lamellipodia) to immature (thin bipolar or tripolar cells) cellular traits (Fig. 3f–i). Thus, the presence of bFGF during the phase of cell expansion fostered a substantial increase in the number of purified iSCs with an immature morphology, Gfap expression and high proliferation rate providing a convenient condition to expand iSCs for multiple passages (tested up to P7) *in vitro*.

### iSCs are efficient in generating myelin *in vitro*

**Figure 4:**
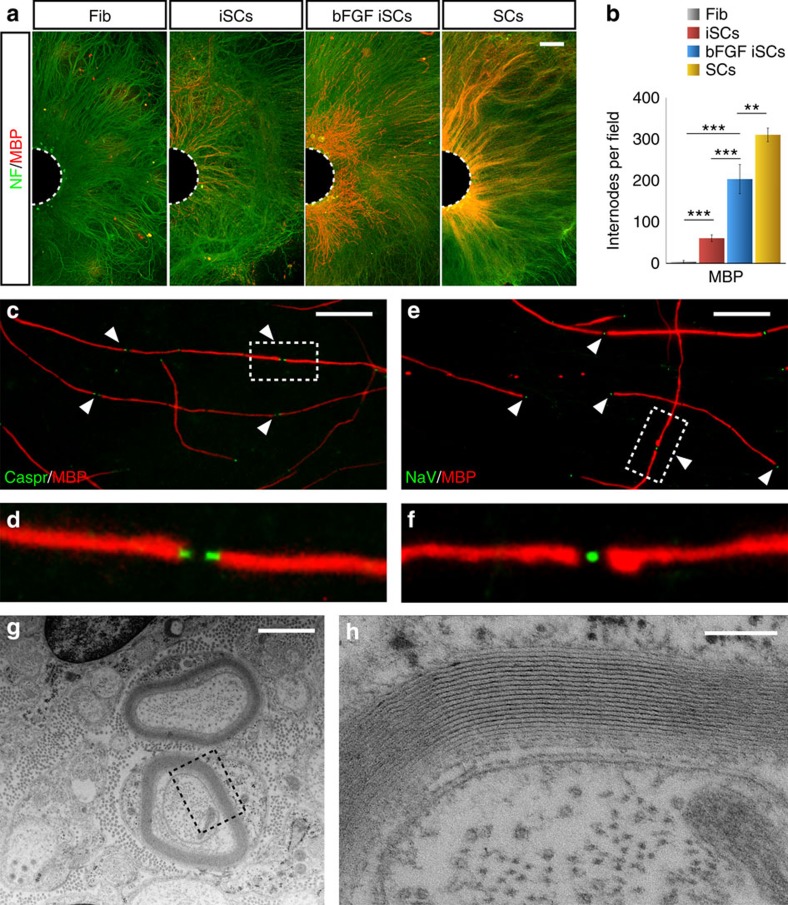
*In vitro* myelination properties of iSCs. (**a**) Myelin generated by fibroblasts (Fib), iSCs with or without bFGF retreatment and somatic SCs plated on mouse DRG axons as visualized by immunofluorescence for MBP (internodes, red), and Neurofilament (NF, green) at 2 weeks of co-culture. (**b**) Quantification of the myelin internodes generated by the four different cells types after 2 weeks of co-culture with DRG neurons (Mean±s.d., *n*=4 independent experiments, 4–8 coverslip/experiment, 10 randomly selected 20 × fields per coverslip were examined). (**c**,**d**) Representative immunofluorescence staining for MPB^+^ internodes together with Caspr (**c**) or NaV (**d**) to visualize the paranodal (arrowsheads) and nodal (arrowheads) regions, respectively, at 4 weeks of iSCs with DRG neurons co-cultures. (**e**,**f**) High-magnification fields of the boxed area in **c**,**d**, respectively. (**g**,**h**) Representative electron microscopy pictures of myelinated iSCs at 4 weeks of co-culture. (**h**) Is a high-magnification image of the boxed area in **e**. **P<0,01; ***P<0,001; One-way ANOVA with Bonferroni correction. Scale bars, 500 μm (**a**), 50 μm (**c**,**d**), 1 μm (**e**), 100 nm (**f**).

To determine the myelinogenic potential of iSCs *in vitro*, we adapted a co-culture system using mouse dorsal root ganglion (DRG) neurons^27^,^28^. DRGs were allowed to develop an extensive axonal network before seeding iSCs pre-treated with or without bFGF. iSCs and DRG co-cultures were maintained for 4 weeks without dox. Intriguingly, in all conditions iSCs, but not fibroblasts, were able to generate Mbp^+^ myelin segments surrounding neuronal axons (Fig. 4a and Supplementary Fig. 7a). However, bFGF-stimulated iSCs produced a three-fold increase in the overall amount of Mbp^+^ myelin segments reaching levels close to those displayed by somatic SCs (Fig. 4b). Likewise also internodal length was comparable between bFGF-stimulated iSCs and primary SCs (Supplementary Fig. 7b). In all conditions, iSC-derived myelin developed mature and well-organized internodes as showed by the correct localization of the paranodal Caspr protein and nodal voltage-gated sodium channels (NaV)^29^ (Fig. 4c–f). Electron microscopy imaging revealed the formation of multiple and compact myelin sheaths around the axons confirming the correct structure of the iSC-derived myelin (Fig. 4g,h). Electron microscopy analyses showed also the presence of non-myelinating iSCs and pre-myelinating iSCs (Supplementary Fig. 7c) indicating that these cells can recapitulate the full behaviour of SCs during myelination. Altogether, these results demonstrate that iSCs acquired a remarkable myelination potential, in particular when previously exposed to exogenous bFGF, generating myelin segments with a correct morphology and organization.

### iSCs from Twicher mice are impaired in myelin formation

**Figure 5:**
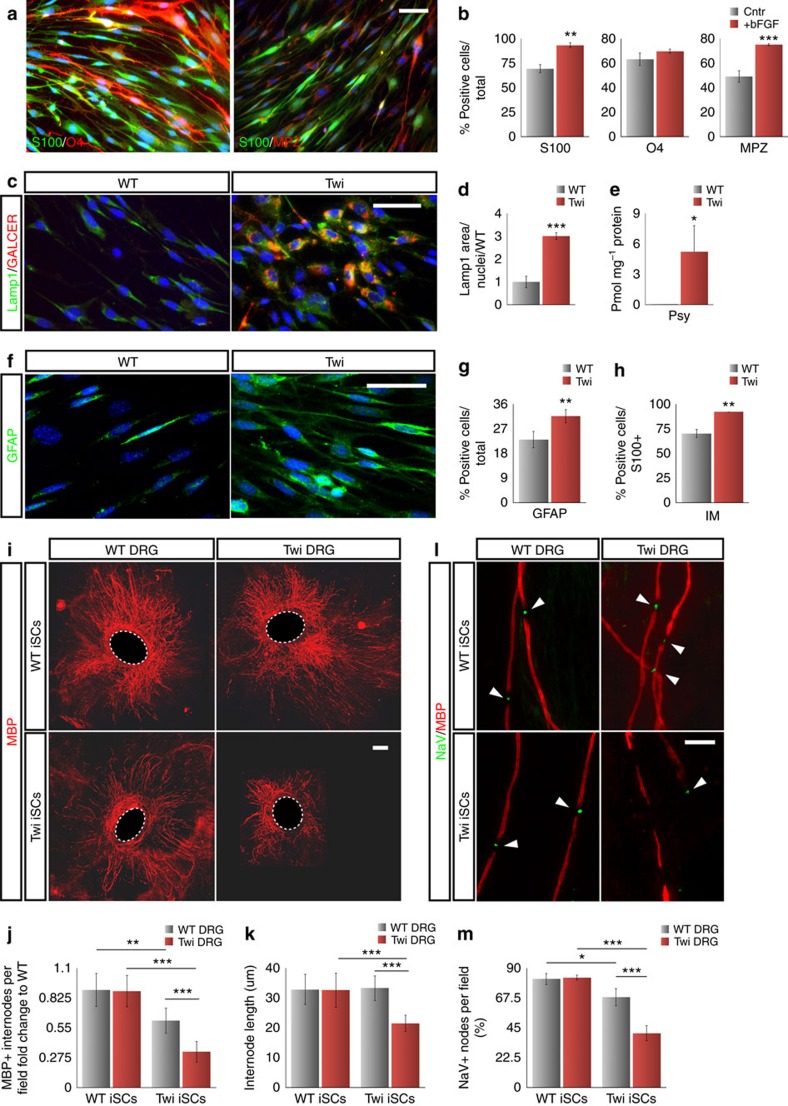
Disease related features of Twitcher (Twi) iSCs and derived myelin. (**a**) Immunofluorescence analysis for S100/O4 and S100/MPZ in O4 immune-purified O4^+^ Twi iSC population reprogrammed in the presence of bFGF. (**b**) Bar graphs showing percentages of S100, O4 and MPZ positive iSCs cells after bFGF treatment or in naive conditions (Mean±s.d., *n*=3 independent experiments, three coverslips/experiment/antigen, 10 randomly selected 20 × fields per sample were examined). (**c**) Representative immunofluorescence pictures for Lamp1 (green) and the lipid metabolite GALCER (red) staining in WT and Twi iSCs. (**d**) Bar graph showing the Lamp1 positive area relative to the total nuclei in Twi iSCs, expressed as fold change (Mean±s.d., WT and Twi *n*=2 independent lines, three coverslips/line, 10 randomly selected 20 × fields per coverslip were examined). (**e**) Mass-spectrometry-based quantification of psychosine (Psy) in WT and Twi iSCs (Mean±s.d., WT and Twi *n*=2 independent lines, three samples per line were examined). (**f**) Representative immunofluorescence images for GFAP in WT and Twi iSCs. (**g**) Quantification of GFAP^+^ cells in WT and Twi iSCs (Mean±s.d., *n*=3 independent experiments, three coverslips/line, 10 randomly selected 20 × fields per sample were examined). (**h**) Quantification of S100^+^ WT and Twi iSCs with immature (IM) morphological traits (Mean±s.d., *n*=3 independent experiments, 10 randomly selected 20 × fields per sample were examined). (**i**) Representative low-magnification pictures of MBP immunostaining to label myelin internodes of iSCs co-cultured with DRG neurons in different combinations between WT and Twi genotypes. (**j**,**k**) Quantification of the number (**j**) and length (**k**) of MBP^+^ internodes in the four different combinations of iSCs and DRG neurons from WT or Twi genotypes (Mean±s.d., *n*=2 independent experiments, 4–8 coverslip/experiment, 10 randomly selected 20 × fields per coverslip were examined). (**l**) Representative immunofluorescence pictures and quantification (**m**) of the NaV^+^ myelin nodes generated after 4 weeks of by co-culture of iSCs with DRG neurons from either WT or Twi genotypes (Mean±s.d., *n*=2 independent experiments, four coverslip/experiment, 10 randomly selected 20 × fields per coverslip were examined). *P<0,05; **P<0,01; ***P<0,001; Student's *t*-test (**b**,**d**,**e**,**h**,**g**); One-way ANOVA with Bonferroni correction (**j**,**k**,**m**). Scale bars, 50 μm (**a**,**c**,**f**); 500 μm (**i**); 20 μm (**l**).

Setting a fast and straightforward method for the generation of myelinating SCs is particularly attractive for *in vitro* disease modelling, since their isolation from mouse tissues and subsequent culture and differentiation is particularly laborious and poorly efficient. Thus, to determine whether iSC reprogramming could represent a useful platform to model PNS pathology *in vitro*, we focused on the well-characterized Twitcher (Twi) mouse model^30^,^31^, which carries a spontaneous mutation inactivating the beta-Galactocerebrosidase (GALC) gene function and closely recapitulates the disease manifestations of the human globoid cell leukodystrophy (GLD, or Krabbe's disease), a severe neurodegenerative lysosomal storage disorder. In GLD, lack of functional GALC results in the accumulation of galactosylceramide (GALCER) and the toxic lysolipid galactosylsphingosine (psychosine) mainly in CNS and PNS myelinating cells, causing progressive demyelination and neurodegeneration^32^,^33^. Nonetheless, models of *in vitro* myelination are not yet available for this disease. Although Twi primary skin fibroblasts displayed some pathological features such as the expansion of the lysosomal compartment and GALCER and psychosine accumulation (Supplementary Fig. 8), they proliferated normally and could be reprogrammed into iSCs at comparable efficiency with their wild-type (WT) counterparts (Supplementary Fig. 9a,b). In addition, upon bFGF treatment Twi and WT iSCs acquired a comparable morphology and growth expansion (Fig. 5a,b and Supplementary Fig. 9c). However, only Twi iSCs displayed significant psychosine accumulation when compared with parental fibroblasts, and evident cytoplasmic deposition of GALCER was observed in Twi iSC as compared with WT counterparts (Fig. 5c–e and Supplementary Fig. 9d,e). Intriguingly, Twi iSCs showed a remarkable immature morphology with a higher number of Ki67^+^ proliferating cells compared with WT counterparts (Fig. 5h and Supplementary Fig. 9f,g). Likewise, a higher number of Twi iSCs upregulated the immature SC marker GFAP while repressing Mbp expression (Fig. 5f,g and Supplementary Fig. 9h,i). These results are in line with a previous study showing that in Twi mice SCs displayed immature features and altered myelin sheath morphology^34^. Next, we assessed the myelinogenic potential of Twi and WT iSCs in organotypic cultures with explanted Twi and WT DRGs in different genotype combinations (Fig. 5i). Intriguingly, co-cultures of both Twi DRG and iSCs revealed a striking loss of MBP^+^ internodes and those remaining exhibited a consistent reduction in average length compared with their counterparts in WT co-cultures at 4 weeks (Fig. 5i, bottom right and top left, Fig. 5j,k and Supplementary Fig. 10). In addition, clustering of sodium channels was impaired suggesting alterations in the nodal structures in Twi iSC-derived myelin (Fig. 5l, bottom right and top left, Fig. 5m). Conversely, in association with Twi DRG, WT iSCs did not reveal any alteration indicating that the loss of GALC enzyme in axons is not affecting the morphological features of the surrounding myelin (Fig. 5i top right and Fig. 5j,k). To determine whether the observed functional defects in Twi SCs could be eventually cross-corrected by WT cells, we co-cultured Twi iSCs with WT DRGs and scored for the presence of myelination defects. Remarkably, in the presence of WT DRGs, the number of Mbp^+^ internodes and their length were significantly normalized and maintained over time in the Twi iSC-derived myelin (Fig. 5i, bottom left, Fig. 5j,k, and Supplementary Fig. 10). Similarly, correct localization of sodium channels at the nodes was strongly ameliorated in these co-cultures (Fig. 5l, bottom left, and Fig. 5m). These results suggest that Twi iSC-related dysfunctions can be largely reverted by WT DRGs. It is well known that lysosomal enzymes are normally released in the extracellular space and can be re-uptaken by neighbour cells (cross-correction)^31^,^35^. Our findings suggest that in our experimental setting the functional GALC enzyme produced by WT DRGs is available for metabolic cross-correction of Twi iSCs. Indeed, previous studies showed that metabolic cross-correction and reconstitution of enzymatic activity mediated by WT NSCs was effective in rescuing critical functions in Twi NSCs and mature progeny *in vitro* and *in vivo*^36^,^37^. Altogether, these results provide evidence that the iSC cellular platform is a powerful and flexible system where investigating pathological mechanisms affecting SC biology and myelination processes.

### Sox10 and Egr2 drive reprogramming into human iSCs

**Figure 6:**
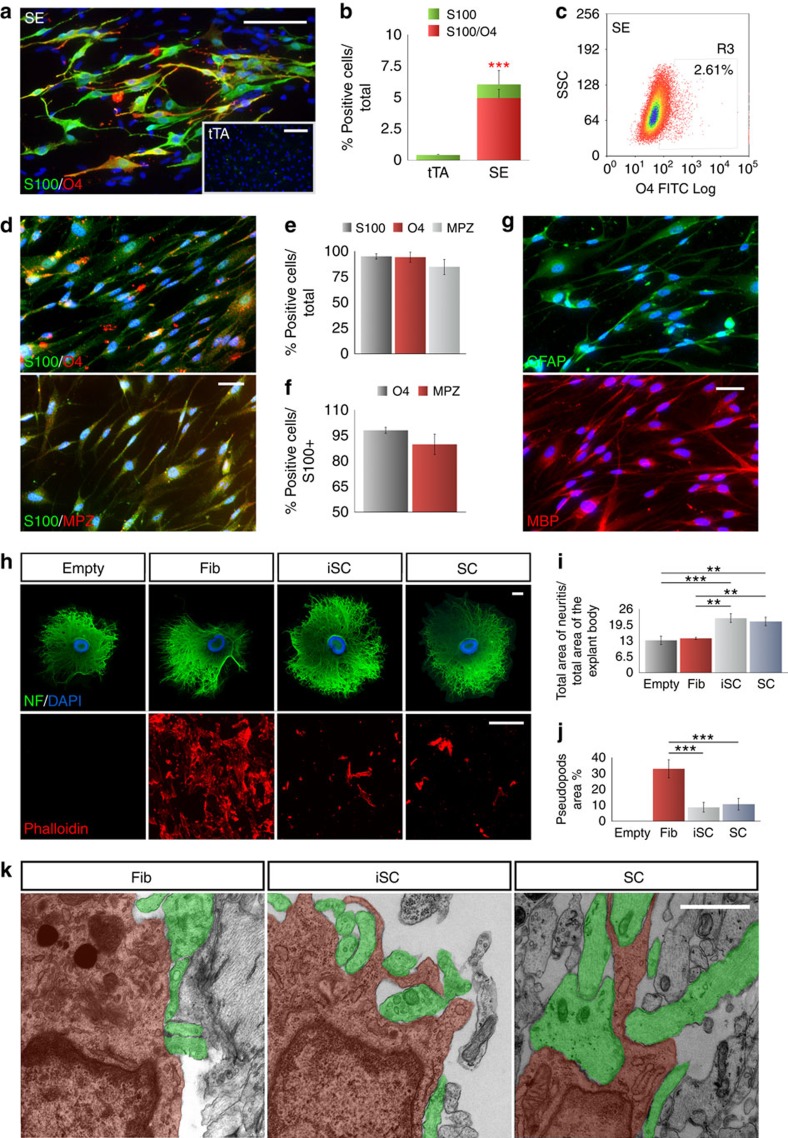
Direct conversion of human skin fibroblasts into iSCs. (**a**) S100 (green) and O4 (red) immunofluorescence staining of Sox10/Egr2 (SE) or tTA-infected (inset) postnatal fibroblasts. (**b**) Quantification of S100^+^ and S100^+^/O4^+^ cells expressed as percentage over the total number of cells in experimental (SE) and control (tTA) conditions at 14 DIV (Mean±s.d., *n*=4 independent experiments, 10 randomly selected 20 × fields per sample were examined). (**c**) Fluorescence-activated cell sorting-based purification of the O4^+^ iSC population. (**d**) S100/O4 and S100/MPZ immunofluorescence staining in iSCs. (**e**,**f**) Quantification of 14 DIV iSCs expressing S100, O4 and MPZ alone or in combination (Mean±s.d., *n*=3 independent experiments, three coverslips/experiment/antigen, 10 randomly selected 20 × fields per sample were examined). (**g**) Immunofluorescence for GFAP and MBP staining. (**h**–**j**) Pseudopod assay: sample images and quantification of DRG axonal growth (**h**, top lane, and **i**) and pseudopod formation (**h**, bottom lane, and **j**) after 5 days of indirect co-culture (Mean±s.d., *n*=3 independent experiments, 2–3 coverslip/experiment, 10 randomly selected 20 × fields per coverslip were examined). (**k**) Representative electron microscopy pictures of one iSC or SC and their pseudopods (brown) contacting multiple axons (green) at 7 days of direct co-culture. **P<0,01; ***P<0,001; Student's *t*-test (**b**); Multiple *t*-test (**i**,**j**). Scale bars, 50 μm (**a**,**d**,**h** bottom); 500 μm (**h**, top); 1 μm (**k**).

Next, we asked whether human fibroblasts of non-neural crest origin could be reprogrammed into SCs. To this end, human postnatal fibroblasts were transduced with Sox10 and Egr2 expressing lentiviruses and cultured for 2 weeks in SC inducing medium. In these conditions, 5±0.6% of the total cell population (*n*=3 independent experiments. Values are means±s.d.) acquired a strong expression of the two SC markers S100 and O4 (Fig. 6a,b). Conversely, no S100^+^/O4^+^ cells were detectable in tTA infected or not infected fibroblast cultures (Fig. 6a, inset). We, then isolated the O4^+^ iSC population by flow-cytometry (Fig. 6c and Supplementary Fig. 11a,b) and grew the cells for 1 week without doxycycline for the following analysis. Fluorescence-activated cell sorting-purified human iSCs (hiSCs) activated several SC markers such as MBP, ERBB3, MAG, EGR2, SOX10 and, most importantly, MPZ (Fig. 6d–f; Supplementary Fig. 11c). Surprisingly, differently from rodent iSCs, hiSCs exhibited a strong expression of both GFAP and MBP (Fig. 6g). As expected, sorted cells did not express oligodendrocyte-specific transcription factors and markers such as MOG, OLIG1 and OLIG2, NG2 and NKX2.2 (Supplementary Fig. 11d), confirming also in this case the PNS regional identity of iSCs.

To functionally characterize hiSCs we adapted the pseudopod assay to study the paracrine effects of iSCs on DRG and *vice versa*^38^. In this indirect co-culture setting SCs were placed over a filter on the top of a Boyden chamber while the DRGs seeded at the bottom of the multiwell (Supplementary Fig. 12a). Three DIV after co-culture, we evaluated the DRG axonal projection area and the pseudopod formation on the internal surface of the Boyden chamber. Interestingly, DRGs, grown in the presence of the hiSCs and primary SC, exhibited significantly longer axons compared with DRGs maintained alone or with control fibroblasts (Fig. 6h, top lane and Fig. 6i), indicating a dual positive paracrine effect between iSCs and DRGs. Furthermore, alike primary SCs and different from fibroblasts, hiSCs failed to extend pseudopods on the internal surface of the Boyden chamber as expected by the distance separating SCs and DRGs which precluded to convey the contact-mediated juxtacrine signals (Fig. 6h, bottom lane and Fig. 6j)^39^. Rat iSCs showed a comparable response in these assays as both hiSC and somatic mouse SCs (Supplementary Fig. 12b–d). Finally, to test the behaviour of the hiSCs to juxtacrine signals, we established direct co-cultures between hiSCs and mouse DRGs. Unfortunately, a reliable protocol for the *in vitro* myelination of hSCs with primary neurons has not yet been developed to date. For these reasons we focused on the early stages of myelination, in which SCs form pseudopods to take direct contact with axons. Interestingly, after 5 DIV of co-culture, electron microscope imaging showed multiple examples of iSCs contacting several axons and emanating pseudopods to surround them, a behaviour normally observed with rodent SCs in the early phase of myelination (Fig. 6k and Supplementary Fig. 12e).

These data clearly indicated that the combination of Sox10 and Egr2 transcription factors is also effective in reprogramming human fibroblasts into induced SCs with an expression pattern and cellular behaviour comparable to their somatic counterpart.

## Discussion

Herein, we identified Sox10 and Egr2 as a unique combination of factors necessary for the effective generation of myelinogenic iSCs by the direct conversion of adult rodent fibroblasts. Remarkably, transcriptome studies revealed a substantial equivalence in the SC-specific molecular signature between somatic and reprogrammed SC progenies with a prevailing epigenetic silencing of the donor cell gene expression profile. Only a minor fraction of specific fibroblast-specific genes (<5%) were found still expressed in iSCs, although at reduced levels compared with the donor cells. This might indicate that 3 weeks of reprogramming in these conditions might not be sufficient to fully reshape the activity of these gene loci. This finding is consistent with previous observations in induced pluripotent stem and fibroblast-to-neuron cell reprogramming, and suggests that the time in culture after reprogramming is a critical factor for the pervasive erasure of the donor cell epigenetic memory^40^,^41^. iSCs showed a robust *in vitro* myelinogenic capacity generating with time correctly structured and patterned myelin sheaths when co-cultured with DRG neurons. To our knowledge, this is the first time that MPZ^+^ SCs with an extremely high myelinating potential are obtained by cell reprogramming. The identification of bFGF as a potent mitogen for iSCs is relevant for enabling the effective expansion of a pure population of iSCs after isolation by immune-purification. Interestingly, initial bFGF treatment also strengthens subsequent iSC differentiation potential and its associated myelin generating capability. This action of bFGF on iSCs is in line with its crucial action in promoting the commitment of immature SCs and maintaining their expansion *in vitro*^25^. Thus, this condition uncovers the opportunity for a large-scale production of iSCs to generate sufficient number of functional iSCs that can be exploited for autologous cell replacement therapies.

The fast and straightforward process to generate iSCs should facilitate *in vitro* disease modelling of peripheral demyelination disorders and axonal damage. In fact, these studies have been hampered by the intrinsic challenge to isolate these cells in high number and sufficient purity, in particular from mice, in which the majority of the genetic models for human diseases are developed. Herein, we provided a strong proof of concept that Twi iSCs retained the cellular and metabolic alterations described in peripheral nerve cells *in vivo,* which result in significant reduced myelination capability and severe alterations in myelin organization^42^,^43^. This setting enabled us to validate a significant rescue of the pathological defects of iSCs by the co-culture with WT DRGs. Further, we provided a direct evidence of a non-cell autonomous mechanism of disease correction likely relying on efficient uptake by Twi iSCs of a functional GALC enzyme produced and secreted by WT DRGs (cross-correction). This phenomenon provides the rationale and is thought to underlie the remarkable beneficial effects of cell and gene therapeutic approaches for lysosomal storage disorders in mice and humans^31^,^44^. However, its direct consequences were not yet corroborated in a physiological *in vitro* model of myelination. Thus, iSCs might be exploited as a useful platform to decipher the molecular mechanisms controlling lysosomal enzymatic release and re-uptake in the contest of normal and pathological SC biology and axonal myelination. More in general, this system can result informative for a broad spectrum of disease conditions.

The iSC direct reprogramming disclosed the opportunity to establish the first strategy of autologous cell transplantation, thereby circumventing immunogenicity, for peripheral nerve injury.

Importantly, the same minimal combination of two factors triggered the conversion of postnatal human fibroblasts into hiSCs. Despite the low reprogramming efficiency, hiSCs could be purified for O4 expression to establish enriched hiSC cultures. Thus, this approach provides a fast and easy protocol to produce hiSCs for biomedical studies. The hurdles experienced in the past in obtaining hSCs have hindered the development of protocols for *in vitro* myelination and *in vivo* transplantation. hiSC generation opens new opportunities to advance experimental methodologies using human cells. This is the first evidence of the generation of reprogrammed SCs endowed with a robust myelinogenic potential suitable for informative *in vitro* studies on physiological and pathological mechanisms of myelin formation/maintenance in peripheral nerves. Therefore, our findings represent a crucial step for the potential future application of iSCs in human PNS disease biology and therapy.

## Methods

### Cell culture

MEFs were isolated from E13.5 wild-type and GALC-deficient Twitcher mice (mixed C57BL6/FVB background; referred to as Twi mice in this study) mice embryos. Head, vertebral column, dorsal root ganglia and all internal organs were removed and discarded and the remaining embryonic tissue was manually minced and incubated in 0.25% trypsin (Sigma) for 10–15 min for cell dissociation. Cells from each embryo were plated onto a 15-cm tissue culture dish in MEF media (Dulbecco's modified Eagle medium (Invitrogen) containing 10% fetal bovine serum (FBS; Hyclone), non-essential amino acids (Invitrogen), L-glutamine (Invitrogen), sodium pyruvate and penicillin/streptomycin (Invitrogen). In all experiments cells were used only within the second passage and not split more than four times. Mouse neonatal and adult fibroblasts and rat neonatal fibroblasts were isolated from tail tip samples. Tails were peeled, minced into 1 mm pieces, placed on culture dishes, and incubated in MEF media for 5 days.

Primary rat SCs were prepared as described^45^ and maintained in SC media (Dulbecco's modified Eagle medium (Invitrogen), 10% FBS (Hyclone), L-glutamine (Invitrogen), 2.5 μM Forskolin (Fsk, Sigma Aldrich), 2.5 ng ml^−1^ recombinant human NRG1β1 (NRG1, EGF domain, R&D), until used.

MRC5 fibroblast cell line was acquired from ATCC (ATCC CCL-171) and maintained in MEF media.

Mouse DRG was isolated from E14.5 wild-type and Twitcher embryos, and established on collagen-coated glass coverslips as described^46^. Explants were cycled with the anti-mitotic reagent 5-fluoro-2-deoxy-uridine to eliminate all non-neuronal cells. Neuronal media was supplemented with 50 ng ml^−1^ NGF (Harlan, Bioproducts for Science) and, in some cases, 25 ng ml^−1^ BDNF (PeproTech) and 10 ng ml^−1^ NT3 (Austral Biologicals). Rat SCs, Fibroblasts and iSCs (200,000 cells/coverslip) were added to established explant cultures of DRG neurons, and myelination was initiated by supplementing media with 50 μg ml^−1^ ascorbic acid (Sigma-Aldrich). Primary human SCs were obtained by Dr K. Haastert-Talini^47^. Testing for *Mycoplasma* cell contamination was routinely performed.

### Molecular cloning and viral infection

Complementary DNAs (cDNAs) for reprogramming transcription factors were cloned into lentiviral vectors under the control of the tetracycline operator. Replication-incompetent, VSVg-coated lentiviral particles were packaged in 293T cells. MEFs, neonatal and adult mouse and neonatal rat fibroblasts were infected in MEF media. Sixteen to twenty hours after infection, cells were switched into fresh MEF media containing doxycycline (2 mg ml^−1^; Sigma). After 48 h medium was replaced with Schwann cell media containing doxycycline. The medium was changed every 2–3 days for a further 12–22 days.

### Immunocytochemical assays

For immunocytochemical analysis, 5 × 10^4^ fibroblasts were plated on matrigel-coated glass coverslips the day before the infection. Ten to twenty-eight days after viral infection cells were fixed for 20 min at room temperature (20–25 °C) in 4% paraformaldehyde in PBS, permeabilized for 30 min in PBS containing 0.1% Triton X-100 and 10% normal goat serum, and incubated overnight at 4 °C in PBS containing 10% normal goat serum and primary antibodies. Then cells were washed three times with PBS and incubated for 2 h at room temperature with secondary antibodies. Co-cultures were permeabilized in methanol and stained as described previously^48^. A list of antibodies used can be found in Supplementary Table 1. The percentages of the cells that were positive to specific markers were determined from at least three independent experiments for each condition. Images for quantification were selected randomly. First, the number of 4,6-diamidino-2-phenylindole-positive nuclei was counted, followed by counting the number of cells expressing the markers of interest in at least five fields per condition. To quantify the immunopositive area (expressed in pixels) pictures in each coverslip/sample were taken and the immunopositive area was calculated using the ImageJ software and normalized on nuclear counts. Cells from WT mice were used to set the signal threshold in the comparison between WT and Twitcher samples.

### RT–PCR analysis

RNA was extracted and purified with the RNeasy Mini kit (QIAGEN). Two micrograms of RNA were reverse transcribed Transcriptor High Fidelity cDNA Synthesis Kit (Roche). One twentieth of the reverse transcribed cDNA was amplified in a 25 microlitres of reaction mixture containing Taq polymerase buffer (Fisher BioReagents), 0.2 mM dNTPs (Finnzymes OY, Espoo, Finland), 0.4 micromolar each primer, 1 U Taq polymerase (Fisher BioReagents). Primers for gene expression analysis are listed in Supplementary Table 2. The thermal profile consisted of a first denaturing step for 5 min at 95 °C, 30 cycles of 30 s at 95 °C, 30 s at the specific annealing temperature (see Supplementary Table 2) and 40 s at 72 °C, followed by a final extension of 10 min at 95 °C.

### RNA sequencing and bioinformatics analysis

Total RNA isolation was performed with the RNeasy Mini Kit (QIAGEN). Libraries were prepared with the TruSeq RNA Library Preparation Kit v2 (Illumina) and sequenced with the Genome Analyser IIx (Illumina). Bioinformatics analysis was performed with the GeneProf web-based software^49^,^50^. Sequence quality control and alignment to the Rn5 reference genome were performed with TopHat 2.1.0 (ref. 51). Estimates of gene expression levels were calculated with the Quantitate Gene Expression module. Differential gene expression was performed with DESeq v2.6 (ref. 52). Statistical analysis was performed with SPSS Statistics (IBM) software and GENE-E (Broad Institute) web-based application. Functional enrichment analysis was performed with FunRich^53^.

### Electron microscopy

Electron microscopy analyses on Schwann cell–DRG neurons myelinating co-cultures were performed as described previously^54^. Cells were fixed in a solution containing 2.5% glutaraldehyde and 0.5% sucrose in 0.1 M Sörensen phosphate buffer (pH 7.2) for 4–6 h. The specimens were then washed in a solution containing 1.5% sucrose in 0.1 M Sörensen phosphate buffer for a period of 6–12 h, post-fixed in 2% osmium tetroxide, dehydrated and embedded in Glauerts' embedding mixture. Semi-thin sections perpendicular were cut on an Ultracut UCT ultramicrotome (Leica, Wetzlar, Germany), and stained with toluidine blue. From the same tissue blocks, ultra-thin sections (50–70 nm) were also cut using the same ultramicrotome and placed on copper grids. Grids were then stained with uranyl acetate and lead citrate and observed on a JEM-1010 transmission electron microscope (JEOL, Tokyo, Japan) operating at 80 kV and equipped with a Mega-View-III digital camera and a Soft-Imaging-System (SIS, Münster, Germany) for the computerized acquisition of the images.

### Determination of GALC activity

Cells were harvested, washed in PBS, lysed for 1 h in 10 mmol l^−1^ sodium phosphate buffer, pH 6.0, containing 0.1% (vol/vol) Nonidet NP-40, and subjected to sonication. These steps were performed at 4 °C. Protein content was measured using the Bradford Protein Assay kit with bovine serum albumin as the reference standard. GALC activity is measured as previously described^55^. Briefly, the assay is performed using 2–7.5 μg of sample proteins with the artificial fluorogenic substrate 4-methylumbelliferone-β-galactopyranoside (1.5 mM) resuspended in 0.1/0.2 mol l^−1^ citrate/phosphate buffer, pH 4.0, and AgNO3 (11 mM) at 37 °C. The enzymatic reaction is stopped by adding 0.2 M pH 10.6 Glycine/NaOH. Fluorescence of liberated 4-methylumbelliferone is measured on a spectrofluorometer (*λ* ex 360 nm, *λ* em 446 nm).

### Psychosine dosage

Psychosine quantification was performed on frozen cellular pellets by Dr W. Kulik (AMC, Amsterdam), as previously described^56^. Briefly, dimethylpsychosine was added to cell-homogenates (0.2–0.5 mg of protein) as internal standard. After deproteinization and extraction by methanol:chloroform and evaporation to dryness of the organic layer, the sample extract was reconstituted in acidic methanol. After injection and elution over a C8 analytical column, psychosine and its internal standard were detected by positive ESI in the MRM mode on a TSQ Quantum AM mass spectrometer. Pychosine concentrations were established by the use of calibration standards.

### Pseudopod assay

Pseudopod assay was performed as previously described^38^. with few modifications: mouse DRG neurons were plated on the bottom of a 24-well plate. Cells (1.5 × 10^5^) were starved for 12 h, and then trypsinized with 0.5% Trypsin–EDTA, pelleted, resuspended and plated into the upper compartment of the Boyden chamber insert. We used 0.33 cm^2^ polycarbonate transwell permeable inserts with 3 μm pores (Corning #3415) coated with matrigel. Cells were maintained in Schwann cell media without doxycycline for 4 h at 37 °C to attach and spread on the upper surface of the insert. Then, inserts were washed with PBS and transferred to a 24-well plate containing DRGs in a medium without ascorbic acid. Pseudopods were allowed to grow at 37 °C. After 3 days, the upper surface of each membrane was cleaned with a cotton swab, and the pseudopods that migrated into the lower surface were fixed in 4% paraformaldehyde and stained with phalloidin (Alexa Fluor 594 phalloidin, Invitrogen). Filters were, next, excised from the insert with microscissors and mounted on a slide for subsequent imaging analysis.

### Animals

Mice were maintained at San Raffaele Scientific Institute Institutional mouse facility (Milan, Italy) in micro-isolators under sterile conditions and supplied with autoclaved food and water. Sprague Dawley rats were housed in the animal facility at the Neuroscience Institute ‘Cavalieri Ottolenghi', c/o Ospedale San Luigi, Orbassano, Turin, Italy. All procedures were performed according to protocols approved by the internal IACUC and reported to the Italian Ministry of Health according to the European Communities Council Directive 2010/63/EU.

### Statistical analysis

Statistical significance between two samples was determined with Student's *t*-test (GraphPad Prism 5; GraphPad). The statistical significance among multiple samples was determined with one-way analysis of variance with Bonferroni's multiple comparison tests. The data were considered statistically significant for *P*<0.05 and are shown as the mean±s.d. or s.e.m as indicated. All data were acquired from at least three independent experiments.

### Data availability

Microarray data have been deposited in the NCBI Gene Expression Omnibus database under accession code GSE78712. The authors declare that all data supporting the findings of this study are available within the article and its Supplementary Information files or from the corresponding author upon reasonable request.

## Additional information

**How to cite this article:** Mazzara, P. G. *et al*. Two factor-based reprogramming of rodent and human fibroblasts into Schwann cells. *Nat. Commun.* **8,** 14088 doi: 10.1038/ncomms14088 (2017).

**Publisher's note**: Springer Nature remains neutral with regard to jurisdictional claims in published maps and institutional affiliations.

## Supplementary Material

Supplementary Information Supplementary Figures and Supplementary Tables
