# Supplementary material for: Two factor-based reprogramming of rodent and human fibroblasts into Schwann cells
Source: Nat Commun. 2017 Feb 7;8:14088. doi: 10.1038/ncomms14088 (PMC5309703; doi:10.1038/ncomms14088)
Supplement: Supplementary Information — Supplementary Figures and Supplementary Tables [file ncomms14088-s1.pdf]

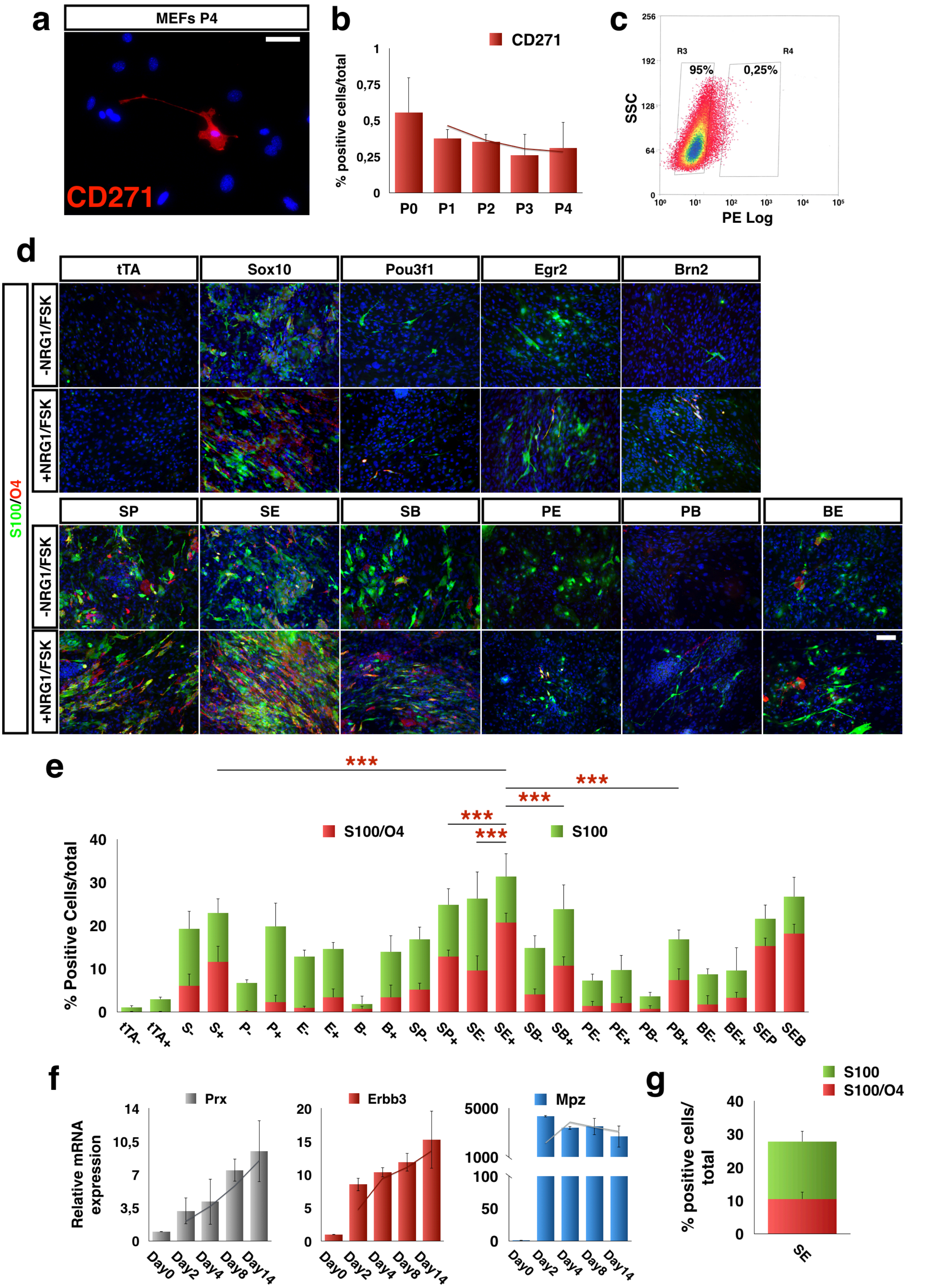

Supplementary Figure 1. Screening for TFs able to convert MEFs into iSCs. (a,b) Representative image (**a**) and quantification (**b**) of CD271 expressing putative neural crest stem cells (red) present in the MEF population (Mean ± SD, n = 5 independent experiments, 10 randomly selected 20x fields per sample were examined). (**c**) FACS plot and gate selection to isolate MEFs lacking $CD271^{+}$ cells. (**d**) Representative S100/O4 immunofluorescence images for the different

combinations of TFs and small molecules for converting MEFs into iSCs. S, Sox10; P, Pou3f1; E, Egr2; B, Brn2. +/- NRG1/Fsk. (**e**) Bar graph comparing the percentage of S100 and S100/O4 positive cells over the total number of plated cells for each reprogramming condition (Mean ± SD, n = 3 independent experiments, 2 coverslips/experiment/antigen, 10 randomly selected 20x fields per sample were examined). (**f**) Expression levels by qRT-PCR analysis over a time of 14 days of reprogramming for the gene Prx, Erbb3 and Mpz (Mean ± SD, n = 3 independent experiments). (**g**) Bar graph showing the percentage of S100 and S100/O4 positive cells over the total number of cells 2 weeks after dox withdrawal (Mean ± SD, n = 3 independent experiments, 2 coverslips/experiment/antigen, 10 randomly selected 20x fields per sample were examined). ***, P<0,001; One-way ANOVA with Bonferroni correction (e). Scale bars: 50 µm (**a**); 100 µm (**d**).

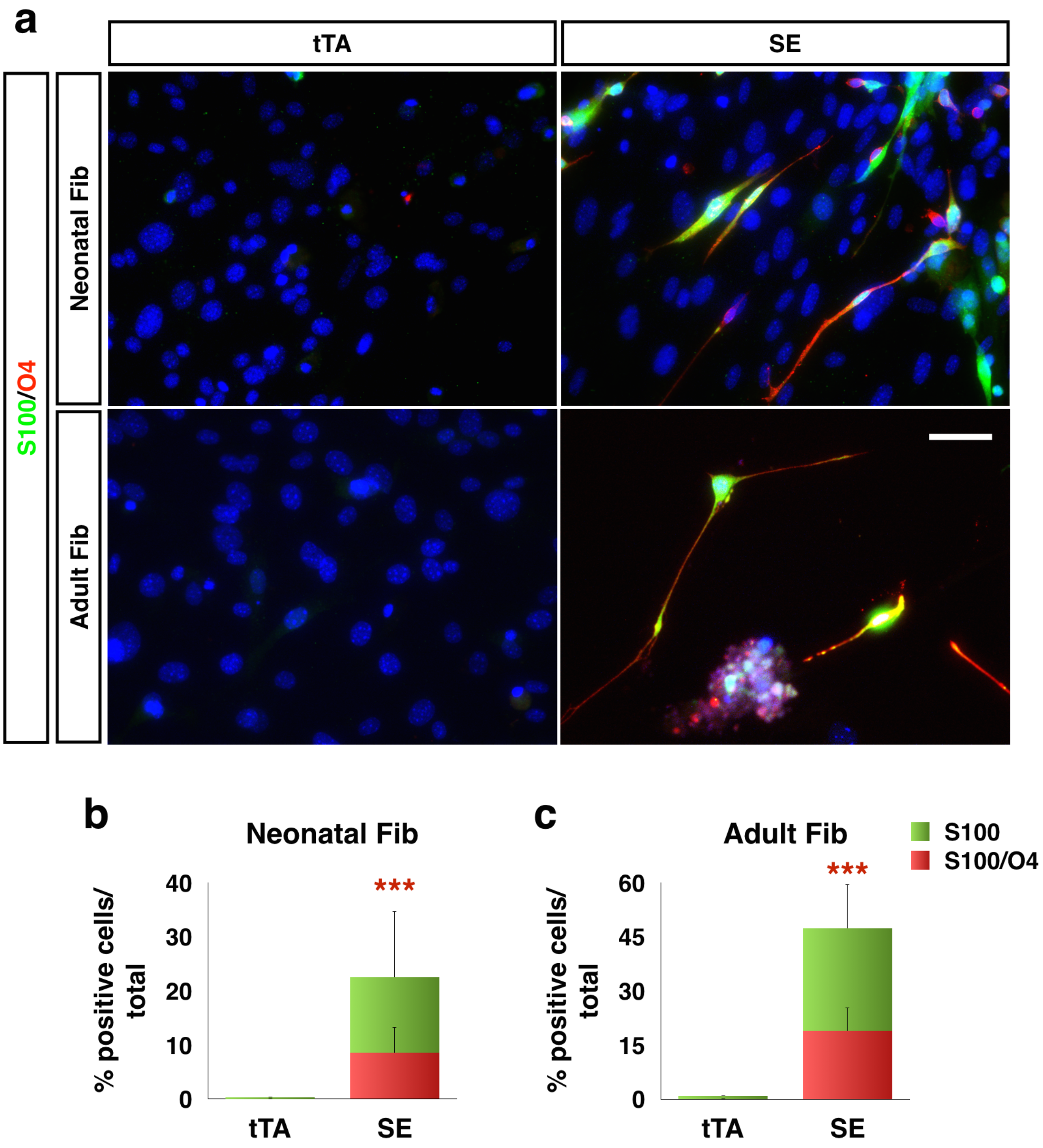

Supplementary Figure 2. Reprogramming of neonatal and adult mouse fibroblasts into iSCs. . (**a**) Representative pictures of S100/O4 immunofluorescence staining of neonatal and adult fibroblasts (Fib) two weeks after infection with Sox10/Egr2 (SE) and tTA (control) expressing lentiviruses. (**b,c**) Quantification of S100 and S100/O4 positive cells on the total number of plated neonatal (**b**) and adult (**c**) fibroblasts two weeks after reprogramming (Mean ± SD, n = 4 independent experiments, 2 coverslips/experiment/antigen, 10 randomly selected 20x fields per sample were examined). ***, P<0,001; Student's t-test (b). Scale bars: 50 µm.

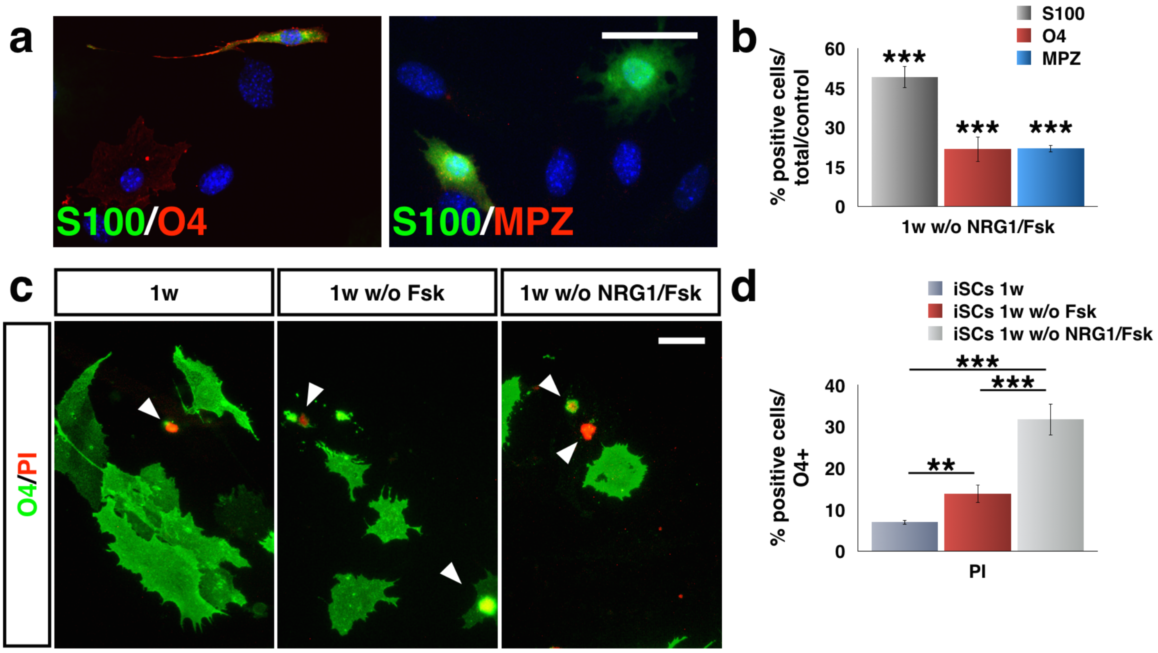

Supplementary Figure 3. Loss of iSCs in absence of NRG1 and Fsk. (**a**) Representative immunofluorescence staining for S100/O4 in iSCs maintained for 1 week in a medium lacking NRG1 and Fsk. (**b**) Percentage of iSCs expressing S100, O4 or MPZ in these culture conditions with or lacking bFGF (Mean ± SD, n = 3 independent experiments, 10 randomly selected 20x fields per sample were examined). Immunofluoresce staining (**c**) and quantification (**d**) of cell death in $O4^+$ iSCs by propidium iodide fluorescent staining in normal culture conditions or 1 week after Fsk withdrawal or NRG1 and Fsk withdrawal (Mean ± SD, n = 3 independent experiments, 2 coverslips/experiment, 10 randomly selected 20x fields per sample were examined). ***, P<0,001; Multiple t-test (b, d). Scale bars: 50 µm.

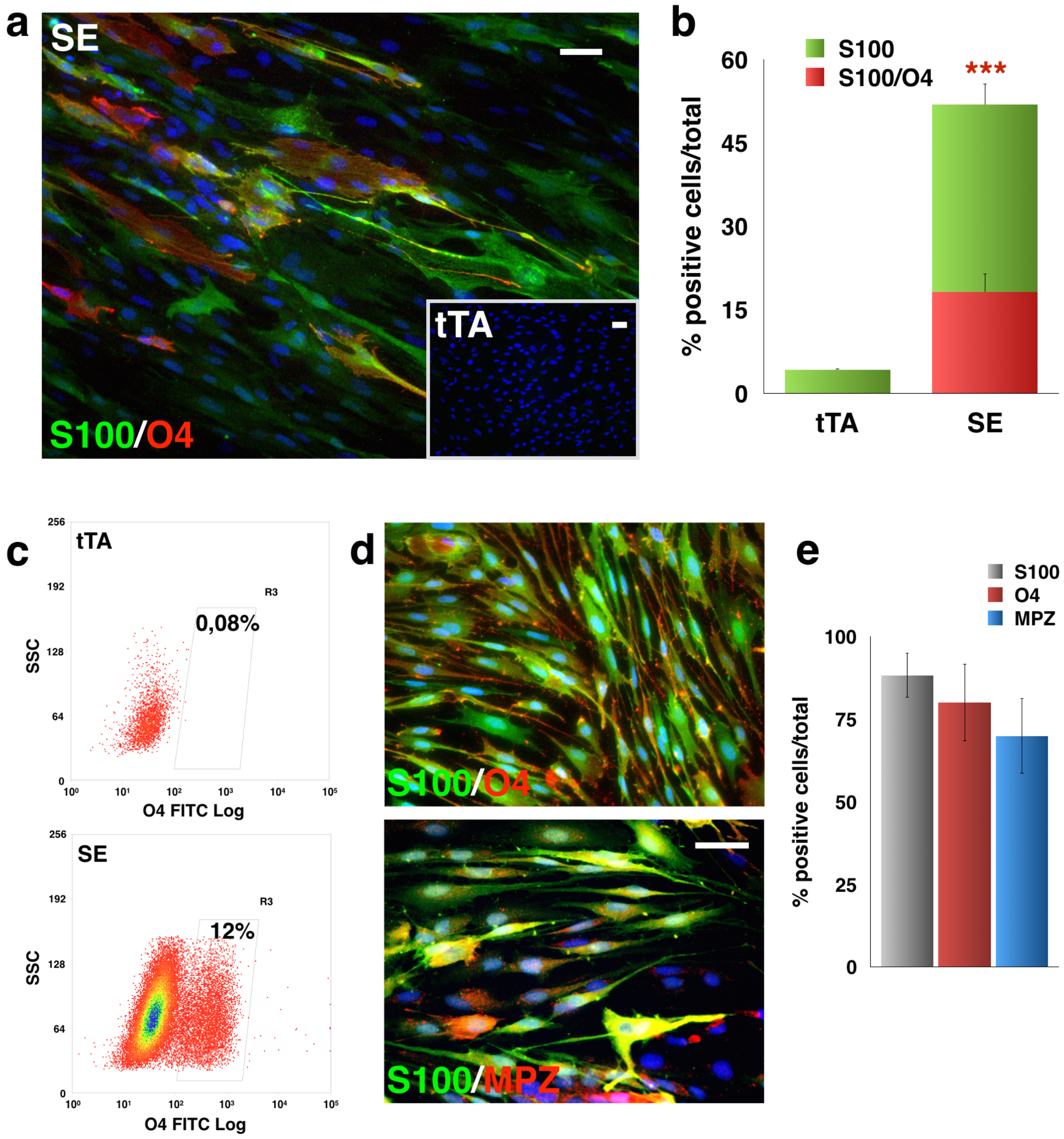

Supplementary Figure 4. Reprogramming of rat juvenile tail fibroblasts into iSCs. (**a**) O4 iSC Representative immunofluorescence staining for S100/O4 on rat tail fibroblasts infected with Sox10/Egr2 (SE) or tTA expressing lentiviruses for 2 weeks. (**b**) Percentage of cells expressing S100 or S100/O4 on the total number of plated cells in the two conditions (Mean ± SD, n = 8 independent rat fibroblast lines, 2 coverslip/experiment/antigen, 10 randomly selected 20x fields per line were examined). (**c**) FACS plots showing the fraction of cells positive for O4 and their quantification after SE or tTA expression. (**d**) Immunofluoresce staining (**d**) and relative quantification (**e**) for S100/O4 and S100/MPZ in O4-purified iSC population during the expansion phase (Mean ± SD, n = 3 independent experiments, 2 coverslips/experiment/antigen 10 randomly selected 20x fields per sample were examined). ***, P<0,001; Student's t-test. Scale bars: 50 µm.

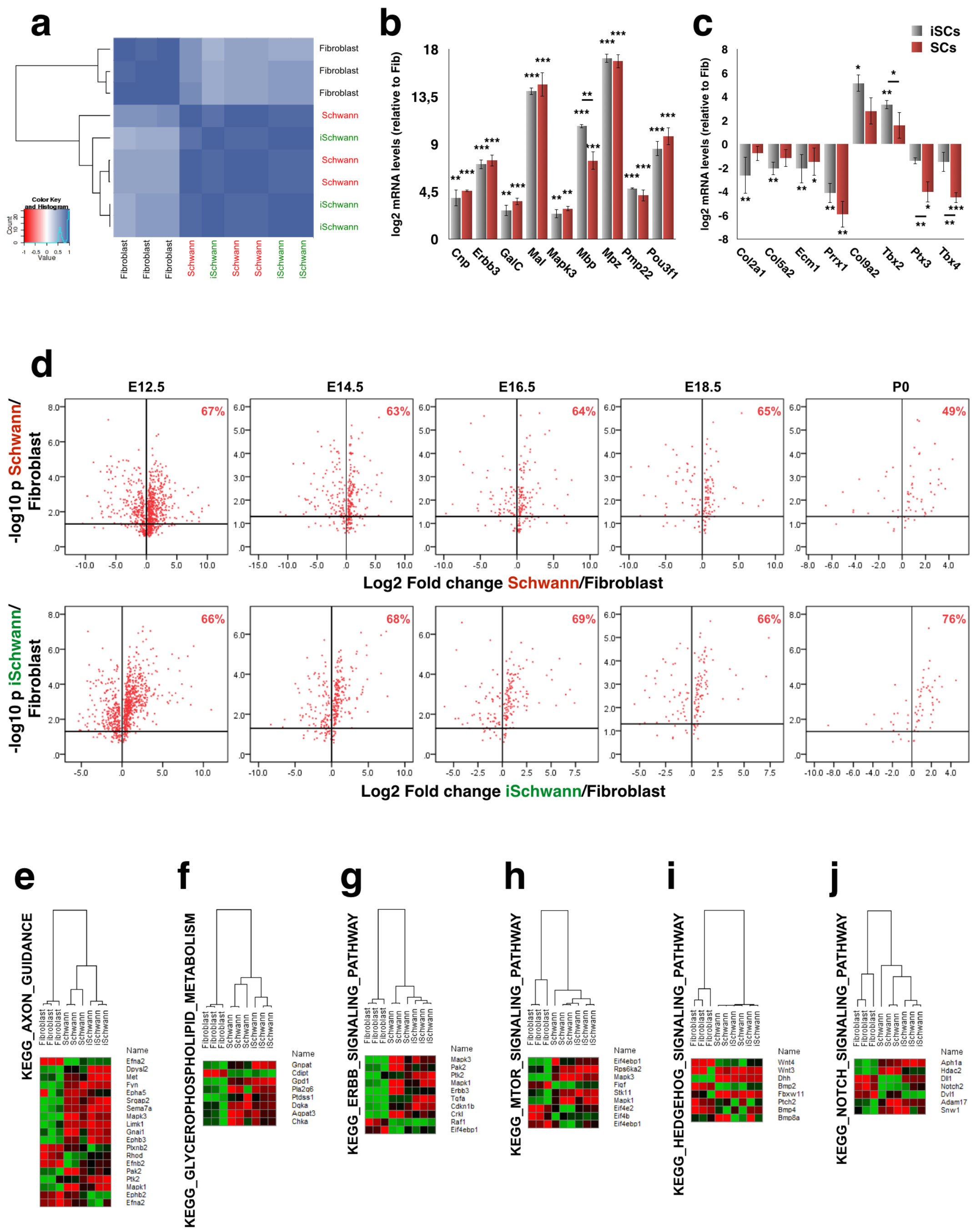

Supplementary Figure 5. Global gene expression profiling of somatic and induced SCs and the fibroblasts of origin. (**a**) Correlation of Expression Patterns in the different samples. The correlation between samples is also shown as a dendrogram on the left. Comparative expression analysis by qRT-PCR analysis for SC-specific (**b**) or fibroblast-specific (**c**) in the three cell types (Mean ± SD, n = 3 independent experiments). (**d**) Volcano plots showing the distribution of the genes up-regulated during the different stages of SC development in the three cell types (Chi square test p=0.39). (**e-j**) Heat-maps showing the expression pattern of the genes belonging to the respective KEGG functional categories in the three cell types. *, P<0,05; **, P<0,01; ***, P<0,001; Multiple t-test (**b, c**)

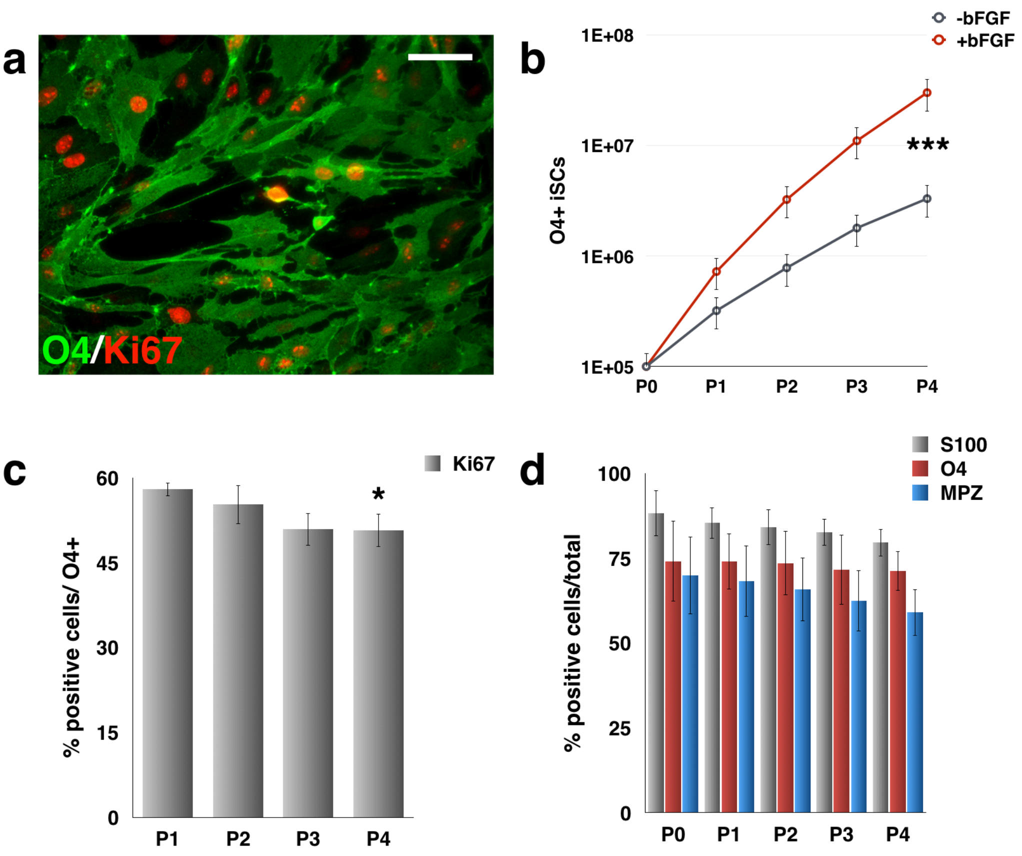

Supplementary Figure 6. Long-term analysis of iSC proliferative ability in the presence of bFGF. (**a**) Representative immunofluorescence staining for Ki67 in $O4^+$ iSCs. (**b**) Proliferation rate of $O4^+$ iSCs after dox withdrawal (P0) in the two bFGF conditions over 4 passages in vitro (about 16 DIV) (Mean ± SD, n = 3 independent experiments were examined). (**c**) Bar graph showing the percentage of $Ki67^+$ cells in the purified $O4^+$ iSC population in the presence of bFGF (Mean ± SD, n = 3 independent experiments, 10 randomly selected 20x fields per sample were examined). (**d**) Quantification of the percentage of iSCs immunodecorated for S100, O4 and MPZ at 4 consecutive passages in culture (Mean ± SD, n = 2 independent experiments, 3 coverslips/experiment/antigen, 10 randomly selected 20x fields per slice were examined). *, P<0,05; ***, P<0,001; Repeated measure Student's t-test. Scale bars: 50 μm.

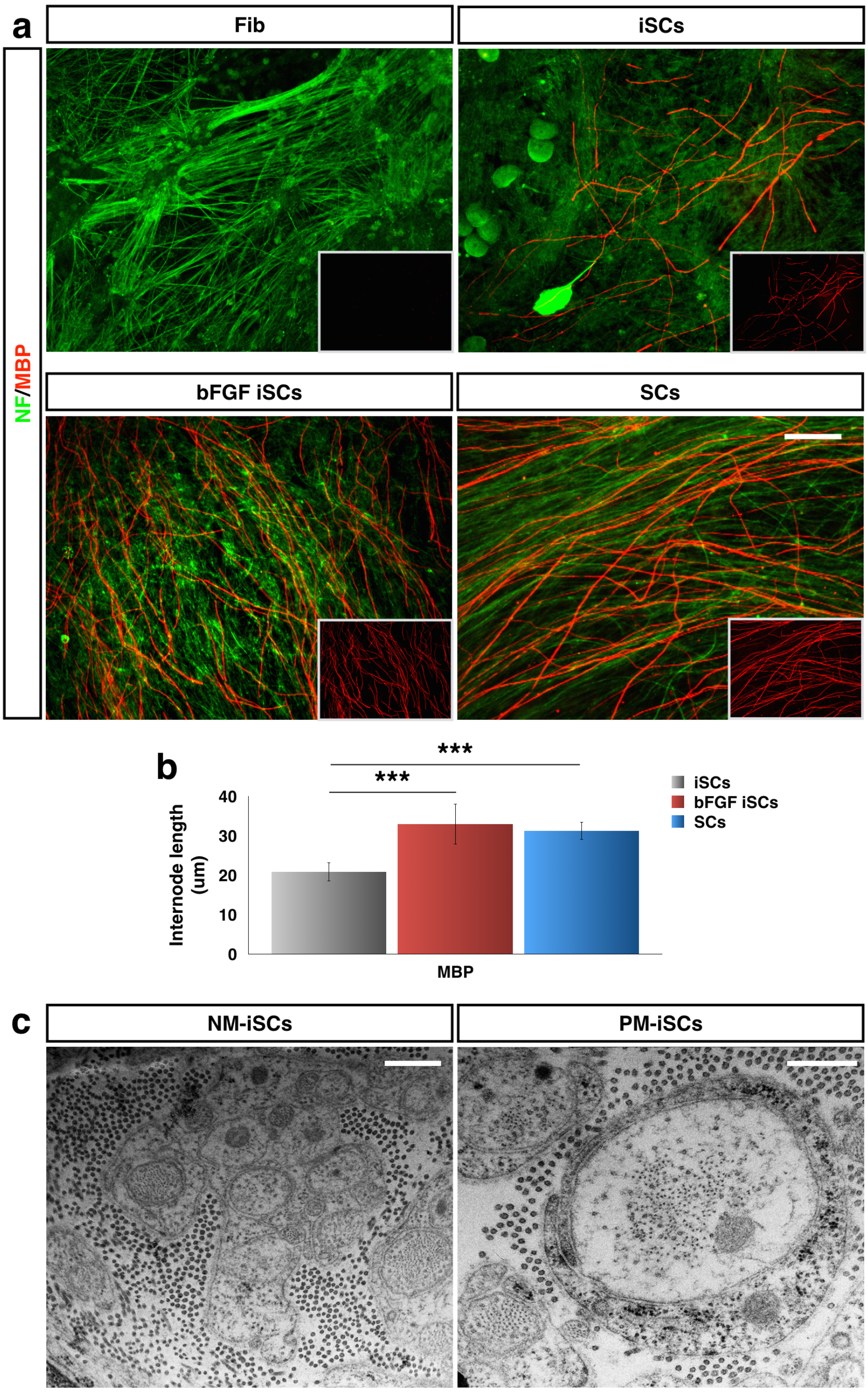

Supplementary Figure 7. *In vitro* myelination of somatic and induced iSCs on DRG axons. (**a,b**) Representative high-magnification images of the immunostaining for MBP/NF (**a**) and quantification of the length of MBP^{+} internodes (**b**) in MEFs (Fib), iSCs, iSCs reprogrammed with bFGF (iSCs +bFGF) and somatic SCs co-cultured for 2 weeks with DRG neurons. (Mean ± SD, n = 4 independent experiments, 4-8 coverslip/experiment, 10 randomly selected 20x fields per coverslip were examined). (**c**) Electron microscopy images of non-myelinating (NM) and pre-myelinating (PM) iSCs at 4 weeks of co-culture with DRG neurons. Scale bars: 100 µm (**a**); 500nm (**c**).

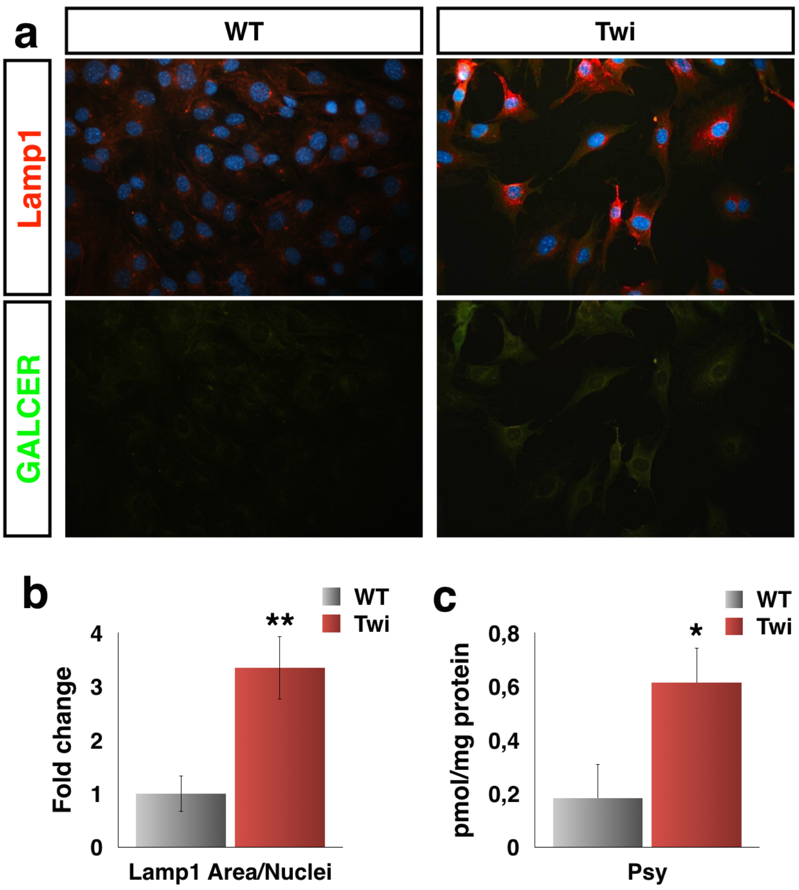

Supplementary Figure 8. *In vitro* analysis of Twitcher (Twi) MEFs. (**a**) Representative immunofluorescence pictures for lysosomal Lamp1 (red) and the lipid metabolite GALCER (green) in WT and Twi MEFs. (**b**) Bar graph showing the Lamp1 positive area relative to the total nuclei in WT and Twi MEFs (Mean ± SD, WT and Twi n = 1 independent line, 2 coverslips/line, 10 randomly selected 20x fields per coverslip were examined). (**c**) Mass-spectrometry-based quantification of psychosine (psy) in WT and Twi MEFs (Mean ± SD, WT and Twi n = 2 independent lines, 2 samples per line were examined). *, P<0,05; **, P<0,01; Student's t-test. Scale bars: 100 µm.

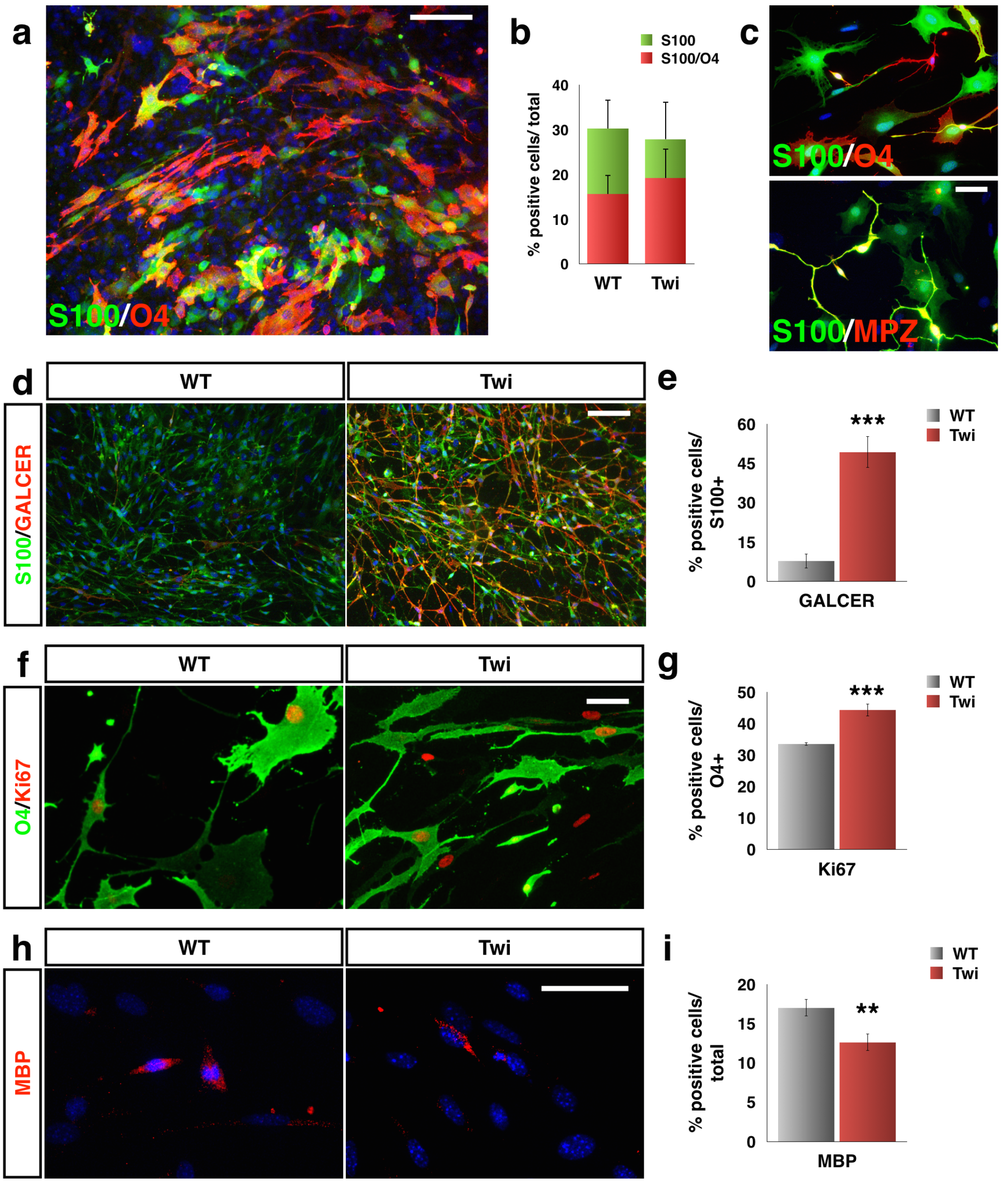

Supplementary Figure 9. Generation and characterization of Twitcher (Twi) iSCs. Immunofluorescence staining (**a**) and quantification (**b**) for S100 and O4 in Twi iSCs 14 days after reprogramming (Mean ± SD, WT and Twi n = 2 independent lines, 3 coverslips/experiment/antigen, 10 randomly selected 20x fields per slice were examined). (**c**) Representative immunofluorescence pictures of double staining for S100/O4 and S100/MPZ in FACS-purified O4^{+} Twi iSCs 1 week after dox withdrawal. (**d**) WT or Twi iSCs immunostained for S100 (green) and the lipid metabolite GALCER (red). (**e**) Bar graph showing the percentage of GALCER^{+} on the S100 expressing cells in WT and Twi iSCs (Mean ± SD, WT and Twi n = 2 independent lines, 2 coverslips/experiment/antigen, 10 randomly selected 20x fields per slice were examined). Immunofluorescence staining (**f**) and quantification (**g**) for O4/KI67 in WT and Twi iSCs (Mean ± SD, n = 3 independent experiments, 2 coverslips/experiment/antigen, 10 randomly selected 20x fields per sample were examined). Immunofluorescence staining (**h**) and quantification (**i**) for MBP in WT and Twi iSCs (Mean ± SD, n = 3 independent experiments, 2 coverslips/experiment/antigen, 10 randomly selected 20x fields per sample were examined). **, P<0,01; ***, P<0,001; Student's t-test. Scale bars: 100 μm (**a**,**d**); 50 μm (**c**,**f**,**h**).

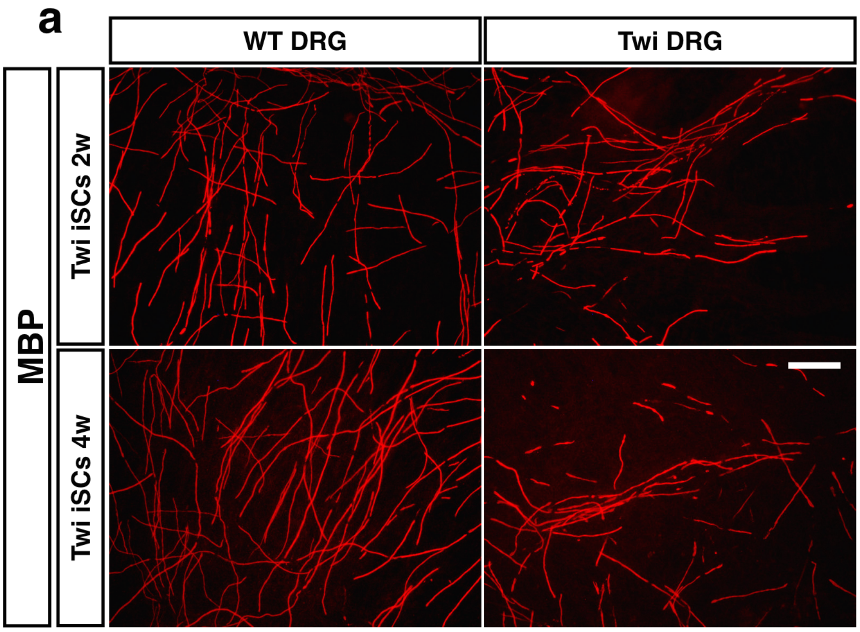

Supplementary Figure 10. Myelin generation in co-cultures between Twi iSCs and WT or Twi DRG neurons at 2 and 4 weeks. (**a**) Representative high-magnification images of MBP immunostaining in co-cultures between Twi iSCs and WT or Twi DRG neurons at 2 (top) and 4 weeks (bottom). Scale bars: 100 µm.

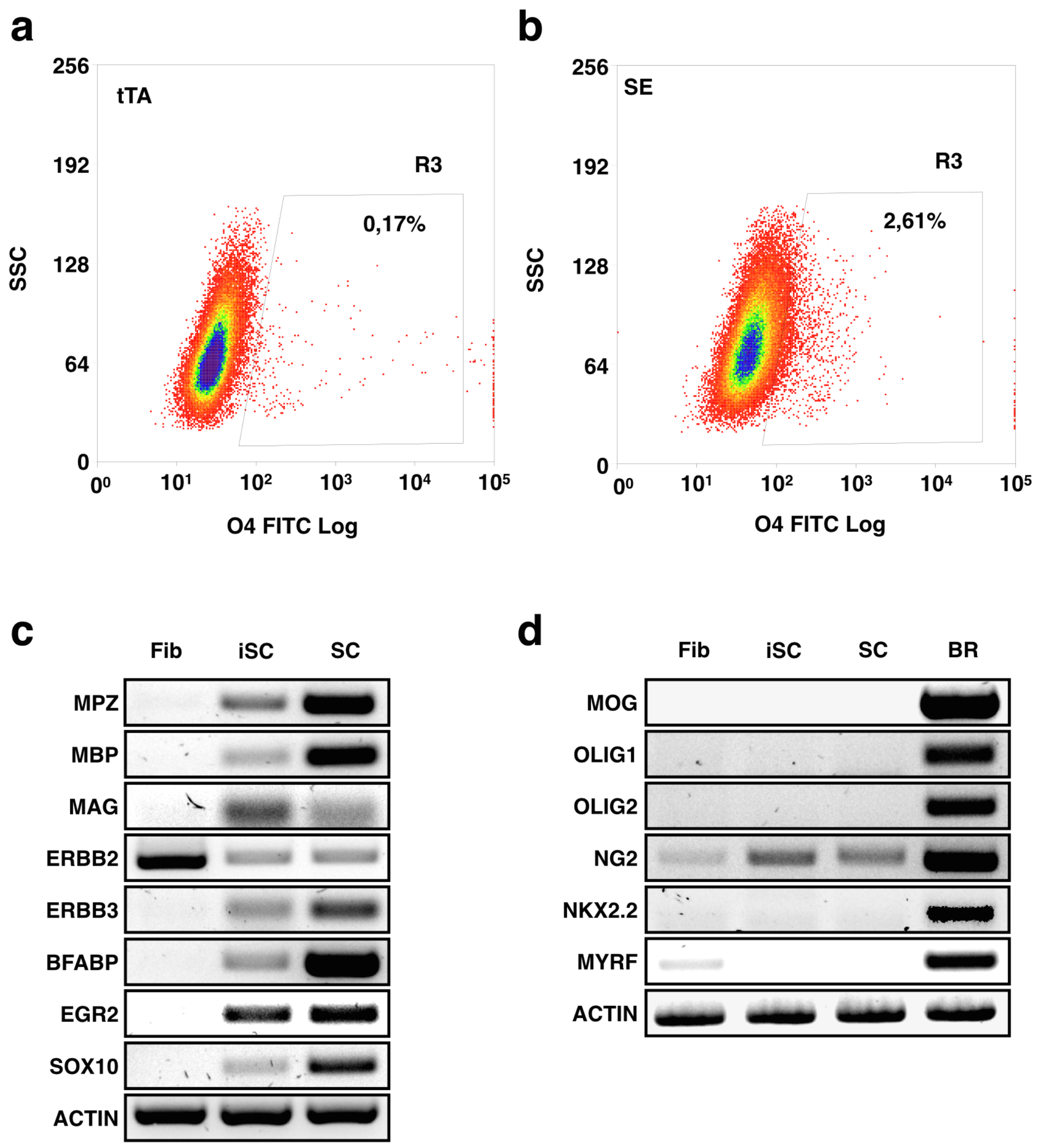

Supplementary Figure 11. FACS-based purification and characterization of the O4$^+$ human iSC population. (**a**) FACS analysis of O4$^+$ cells in tTA control group. (**b**) FACS analysis of O4$^+$ cells in the Sox10/Egr2 experimental group. (**c**) Expression of SC cardinal markers by RT-PCRs in fibroblasts (Fib), induced SCs and human Schwann cells (SC, positive control). (**d**) Expression of oligodendrocyte specific markers by RT-PCRs in fibroblasts (Fib), induced SCs, adult human Schwann cells (SC) and adult human brain (BR, positive control).

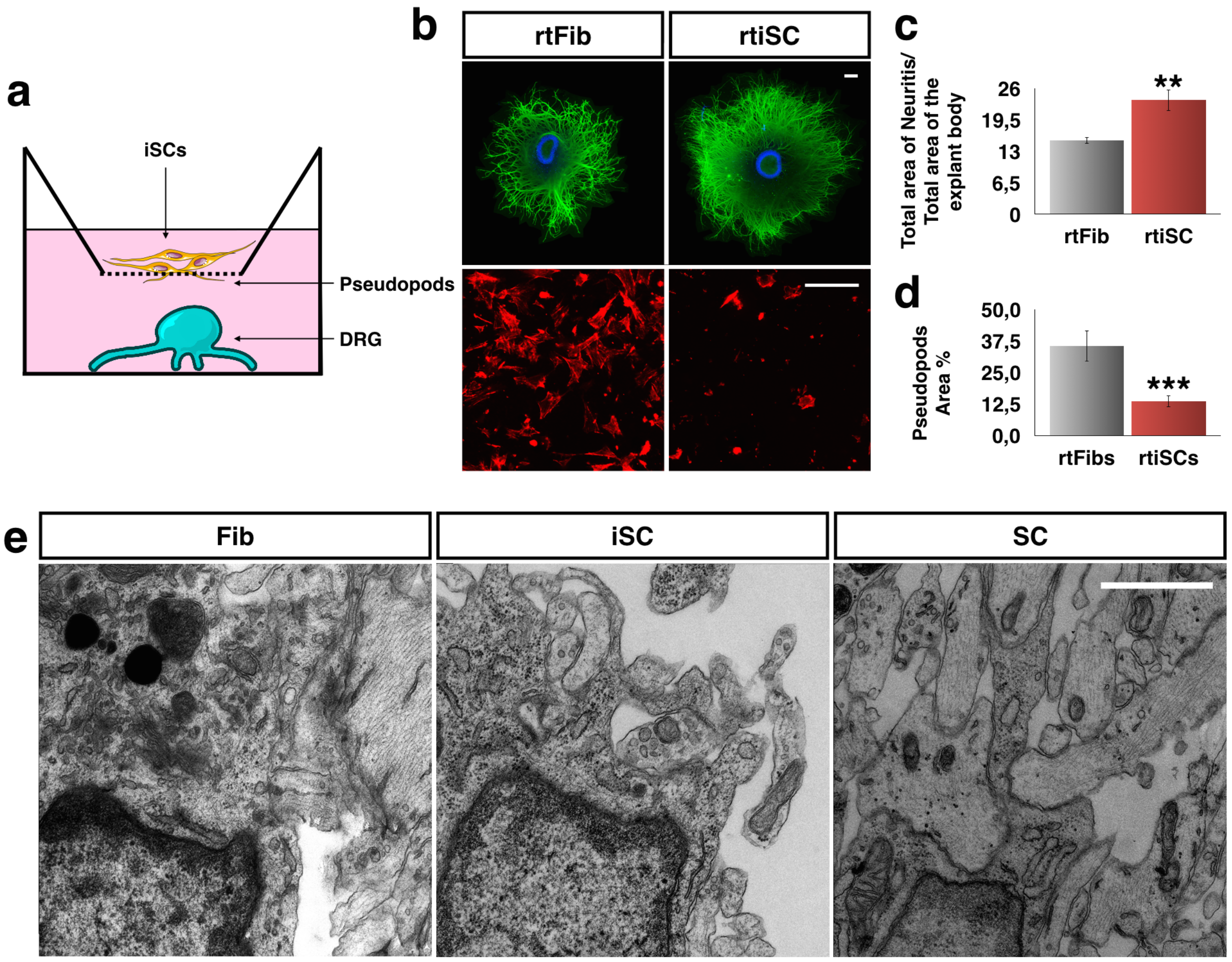

Supplementary Figure 12. Functional assessment of human iSCs. (**a**) Schematic representation of pseudopod assay: DRGs were plating on the bottom of the multiwell plate; Fibroblasts, iSCs or primary SCs were plating on the upper side of the Boyden chamber with microporous filters (3 µm pores). Pseudopods formation was evaluated analysing the bottom surface of the Boyden chamber. (**b-d**) Pseudopods assay performed on rat Fibroblasts (rtFib) and rat iSCs (rtiSC) as controls: sample images and quantification of DRG growth (**b**, top lain, and **c**) and pseudopods formation (**b**, bottom lain, and **d**) after 3 days of indirect co-culture. (**e**) Original electron microscopy pictures presented with a superimposed colour code in **Fig. 7k**. **, P<0,01; ***, P<0,001; Student's t-test (**c**,**d**). Scale bars: 50 µm (**b**, bottom); 500 µm (**b**, top); 1 µm (**e**).

Supplementary Table 1:

Oligonucleotides used for the amplification of the indicated genes.

Gene	Primer sequence (5'-3')	Annealing T°
human ACTIN Fw	ACCCCAGCCATGTACGTT	61
human ACTIN Rev	GGTGAGGATCTTCATGAGGTAG	61
human BFABP Fw	GCACATTCAAGAACACGGAGA	61
human BFABP Rev	CACATCACCAAAAGTAAGGGTCA	60
human EGR2 Fw	TCCTGTCCCTCTCTCCCTTT	60
human EGR2 Rev	CCTTTGCCTTGGGTTGATAG	60
human ERBB2 Fw	TGTGACTGCCTGTCCCTACAA	61
human ERBB2 Rev	CCAGACCATAGCACACTCGG	60
human ERBB3 Fw	GGTGATGGGGAACCTTGAGAT	61
human ERBB3 Rev	CTGTCACTTCTCGAATCCACTG	60
human MAG Fw	CCAAGTAGTCCACGAGAGCTT	61
human MAG Rev	CAGGTCCCCACGGAAGTAGT	62
human MBP Fw	GTCCCTGAGCAGATTTAGCTG	60
human MBP Rev	GAATCCCTTGTGAGCCGATTT	60
human MOG Fw	ACCAGGCACCTGAATATCGG	61
human MOG Rev	CAGGGCTCACCCAGTAGAAAG	61
human MPZ Fw	CATCGTGGTTTACACCGACAG	61
human MPZ Rev	TGGAAGATCGAAATGGCATCTCT	61
human MYRF Fw	AACATGCGTAAGAAGGGCAAG	61
human MYRF Rev	CCAGCGTGTAGTTCTGGTTC	61
human NG2 Fw	CTTTGACCCTGACTATGTTGGC	61
human NG2 Rev	TGCAGGCGTCCAGAGTAGA	62
human NKX2.2 Fw	CCGGGCCGAGAAAGGTATG	62
human NKX2.2 Rev	GTTTGCCGTCCCTGACCAA	62
human OLIG1 Fw	AAAGTGACCAGAGCGGATGT	60
human OLIG1 Rev	CAACTCCAGGGACAAGGAGA	60
human OLIG2 Fw	TGGCTTCAAGTCATCCTCGTC	62
human OLIG2 Rev	ATGGCGATGTTGAGGTCGTG	62
human SOX10 Fw	ATCCAGGCCCACTACAAGAG	59
human SOX10 Rev	GAAGTCGATGTGAGGCTTCC	59
mouse Cnx32 Fw	GCACGTAGCTCACCAACAG	60
mouse Cnx32 Rev	TGATGACATAGGTCCACCACA	60
mouse Egr2 endo Fw	CTACCACCCTTCCCTGTTCC	60
mouse Egr2 endo Rev	AACTCCCTTCAAATGGCCTAA	60
mouse Erbb2 Fw	ACCGACATGAAGTTGCGACTC	61
mouse Erbb2 Rev	AGGTAAGCTCCAAATTGCCCT	61
mouse Erbb3 Fw	AAGTGACAGGCTATGTACTGGT	60
mouse Erbb3 Rev	GCTGGAGTTGGTATTGTAGTTCA	60

mouse Mag Fw	CGAGGGTTACGCCAGTTT	59
mouse Mag Rev	ATCCCGCATCCAAGTCAG	59
mouse Mbp Fw	AATCGGCTCACAAGGGATTCA	60
mouse Mbp Rev	TCCTCCCAGCTTAAAGATTTTGG	60
mouse Mog Fw	ACCTCTACCGAAATGGCAAGG	61
mouse Mog Rev	TCACGTTCTGAATCCTAAGGGT	61
mouse Mpz Fw	TACAGTGACAACGGCACTTTC	61
mouse Mpz Rev	GCAGTACCGAATCAGGTAGAAGA	61
mouse Myrf Fw	TCTGGGCCTCCCATCAAAG	61
mouse Myrf Rev	CGGGGTTATGGTGCGTAGAAG	61
mouse Ng2 Fw	GCTGTCTGTTGACGGAGTGTT	62
mouse Ng2 Rev	CGGCTGATTCCCTTCAGGTAAG	62
mouse Nkx2.2 Fw	ACAACCCCTACACTCGCTG	61
mouse Nkx2.2 Rev	GTCATTGTCCGGTGACTCGTC	62
mouse Olig1 Fw	GCAGCCACCTATCTCCTCATC	61
mouse Olig1 Rev	CGAGTAGGGTAGGATAACTTCGC	61
mouse Olig2 Fw	GGCGGTGGCTTCAAGTCAT	62
mouse Olig2 Rev	CATGGCGATGTTGAGGTCG	61
mouse Pmp22 Fw	ATGGACACACGACTGATCTCT	60
mouse Pmp22 Rev	CAGCCATTCGCTCACTGATGA	62
mouse Sox10 endo Fw	CTACGACTGGACGCTGGTG	61
mouse Sox10 endo Rev	GTGAGGGTACTGGTCGGCTA	61
mouse/rat 18S Fw	GTAACCCGTTGAACCCCATT	61
mouse/rat 18S Rev	CCATCCAATCGGTAGTAGCC	61
rat Cnp Fw	AGCAGAGGAGGAGGTGGATGT	61
rat Cnp Rev	GCAGGAGGGCACTGGACA	61
rat Col1a2 Fw	TCGAGACCCTTCTCACTCCT	60
rat Col1a2 Rev	GCATCCATAGTGCATCCTTG	60
rat Col5a2 Fw	AATTGTAACGGGCATACGTG	59
rat Col5a2 Rev	ACACCTGGCTCTCCATCAAT	59
rat Col9a2 Fw	TCCCTGGTGAGATTGGAAC	59
rat Col9a2 Rev	AGGACAGTTGGTTGGACACA	59
rat Ecm1 Fw	GATCGGGAGCTGGCTATAAA	59
rat Ecm1 Rev	GGTCAACGGTCAAGAGATCC	59
rat Erbb3 Fw	CCCTATGCAGGGTTACGACT	59
rat Erbb3 Rev	CAATCATCCAGCACTTGACC	59
rat Galc Fw	CCCTTCCCAACCAGCTATAA	59
rat Galc Rev	AAGCGATGCTCAAGGTCTTC	59
rat Mal Fw	TTTACCTCAGTGCCTCAGTCC	59
rat Mal Rev	GCATGGATCACGTAGAGCAG	59
rat Mapk3 Fw	AAGGCCCGAAACTACCTACA	59
rat Mapk3 Rev	TACTGTGATGCGCTTGTTTG	59
rat Mbp Fw	ACACACAAGAACTACCCACTACGG	61

rat Mbp Rev	GTACGAGGTGTCACAATGTTCTTG	61
rat Mpz Fw	CACAACCTAGACTACAGTGACAACG	61
rat Mpz Rev	TTCGAGGAGTCCTTAGAAGACTTG	61
rat Pmp22 Fw	TCTGTCATCTTCAGCGTCCT	59
rat Pmp22 Rev	GCTGCACTCATCACACACAG	59
rat Pou3f1 Fw	AAACCGCTGCTCAACAAGT	59
rat Pou3f1 Rev	CCTTTGACACCCACCTCAAT	59
rat Prrx1 Fw	AGAACCGAAGAGCCAAGTTC	59
rat Prrx1 Rev	GGAGCAGGACGAGGTACAAT	59
rat Ptx3 Fw	GGCTTCAATATCTGGGATCG	60
rat Ptx3 Rev	TCCTCCATGAGCCTGAATCT	60
rat Tbx2 Fw	GAAGATCGACAACAACCCTTT	60
rat Tbx2 Rev	CTCCGGTTTACAGTGCTCCT	60
rat Tbx4 Fw	GACAACAAATGGATGGTTGC	59
rat Tbx4 Rev	GCTTCAGCTTCTGGAAAGAGA	59

Supplementary Table 2:

List of primary antibodies used for immunofluorescence.

Target	Species	Diluition	Company
Caspr	mouse	1:100	NeuroMab
CD271	rabbit	1:100	Promega
CD271-PE	mouse	1:100	Milteny
Galcer	mouse	1:300	Millipore
GFAP	rabbit	1:100	Dako
Ki67	rabbit	1:1000	Immunological Science
Lamp1	rabbit	1:200	Abcam
MBP	mouse	1:1000	Covance
MBP hybridoma	Rat	1:2	Home made
MPZ	chicken	1:100	Aves
Nav	mouse	1:500	Sigma
NF	chicken	1:2000	Covance
O4 hybridoma	mouse	1:2	Home made
S100	rabbit	1:500	Dako
